# Loss of UGP2 in brain leads to a severe epileptic encephalopathy, emphasizing that bi-allelic isoform-specific start-loss mutations of essential genes can cause genetic diseases

**DOI:** 10.1007/s00401-019-02109-6

**Published:** 2019-12-09

**Authors:** Elena Perenthaler, Anita Nikoncuk, Soheil Yousefi, Woutje M. Berdowski, Maysoon Alsagob, Ivan Capo, Herma C. van der Linde, Paul van den Berg, Edwin H. Jacobs, Darija Putar, Mehrnaz Ghazvini, Eleonora Aronica, Wilfred F. J. van IJcken, Walter G. de Valk, Evita Medici-van den Herik, Marjon van Slegtenhorst, Lauren Brick, Mariya Kozenko, Jennefer N. Kohler, Jonathan A. Bernstein, Kristin G. Monaghan, Amber Begtrup, Rebecca Torene, Amna Al Futaisi, Fathiya Al Murshedi, Renjith Mani, Faisal Al Azri, Erik-Jan Kamsteeg, Majid Mojarrad, Atieh Eslahi, Zaynab Khazaei, Fateme Massinaei Darmiyan, Mohammad Doosti, Ehsan Ghayoor Karimiani, Jana Vandrovcova, Faisal Zafar, Nuzhat Rana, Krishna K. Kandaswamy, Jozef Hertecant, Peter Bauer, Mohammed A. AlMuhaizea, Mustafa A. Salih, Mazhor Aldosary, Rawan Almass, Laila Al-Quait, Wafa Qubbaj, Serdar Coskun, Khaled O. Alahmadi, Muddathir H. A. Hamad, Salem Alwadaee, Khalid Awartani, Anas M. Dababo, Futwan Almohanna, Dilek Colak, Mohammadreza Dehghani, Mohammad Yahya Vahidi Mehrjardi, Murat Gunel, A. Gulhan Ercan-Sencicek, Gouri Rao Passi, Huma Arshad Cheema, Stephanie Efthymiou, Henry Houlden, Aida M. Bertoli-Avella, Alice S. Brooks, Kyle Retterer, Reza Maroofian, Namik Kaya, Tjakko J. van Ham, Tahsin Stefan Barakat

**Affiliations:** 1 Department of Clinical Genetics, Erasmus MC University Medical Center, Rotterdam, The Netherlands; 2 Department of Genetics, King Faisal Specialist Hospital and Research Centre, Riyadh, 11211 Kingdom of Saudi Arabia; 3 Department for Histology and Embryology, Faculty of Medicine Novi Sad, University of Novi Sad, Novi Sad, Serbia; 4 iPS Cell Core Facility, Erasmus MC University Medical Center, Rotterdam, The Netherlands; 5 Department of (Neuro)Pathology, Amsterdam Neuroscience, Amsterdam UMC, University of Amsterdam, Amsterdam, The Netherlands; 6 Stichting Epilepsie Instellingen Nederland (SEIN), Zwolle, The Netherlands; 7 Center for Biomics, Department of Cell Biology, Erasmus MC University Medical Center, Rotterdam, The Netherlands; 8 Department of Neurology, Erasmus MC University Medical Center, Rotterdam, The Netherlands; 9 Division of Genetics, McMaster Children’s Hospital, Hamilton, ON L8S 4J9 Canada; 10 Division of Cardiovascular Medicine, Stanford University School of Medicine, Stanford, CA 94035 USA; 11 Division of Medical Genetics, Department of Pediatrics, Stanford University School of Medicine, Stanford, CA 94035 USA; 12 GeneDx, Gaithersburg, MD 20877 USA; 13 Department of Child Health, College of Medicine and Health Sciences, Sultan Qaboos University, Muscat, Oman; 14 Genetic and Developmental Medicine Clinic, Sultan Qaboos University Hospital, Muscat, Oman; 15 Department of Radiology and Molecular Imaging, Sultan Qaboos University Hospital, Muscat, Oman; 16 Department of Human Genetics, Radboud University Medical Centre, Nijmegen, The Netherlands; 17 Department of Medical Genetics, Faculty of Medicine, Mashhad University of Medical Sciences, Mashhad, Iran; 18 Medical Genetics Research Center, Mashhad University of Medical Sciences, Mashhad, Iran; 19 Genetic Center of Khorasan Razavi, Mashhad, Iran; 20 Student Research Committee, Faculty of Medicine, Mashhad University of Medical Sciences, Mashhad, Iran; 21 Genetic Counseling Center, Welfare Organization of Sistan and Baluchestan, Zahedan, Iran; 22 Department Medical Genetics, Next Generation Genetic Polyclinic, Mashhad, Iran; 23 Molecular and Clinical Sciences Institute, St. George’s University of London, Cranmer Terrace, London, SW17 0RE UK; 24 Innovative Medical Research Center, Mashhad Branch, Islamic Azad University, Mashhad, Iran; 25 Department of Neuromuscular Disorders, UCL Queen Square Institute of Neurology, London, WC1N 3BG UK; 26 Department of Paediatric Neurology, Children’s Hospital and Institute of Child Health, Multan, 60000 Pakistan; 27 CENTOGENE AG, Rostock, Germany; 28 Department of Pediatrics, Tawam Hospital, and College of Medicine and Health Sciences, UAE University, Al-Ain, UAE; 29 Department of Neurosciences, King Faisal Specialist Hospital and Research Centre, Riyadh, 11211 Kingdom of Saudi Arabia; 30 Neurology Division, Department of Pediatrics, College of Medicine, King Saud University, Riyadh, 11461 Kingdom of Saudi Arabia; 31 Department of Pathology and Laboratory Medicine, King Faisal Specialist Hospital and Research Centre, Riyadh, 11211 Kingdom of Saudi Arabia; 32 Radiology Department, King Faisal Specialist Hospital and Research Centre, Riyadh, 11211 Kingdom of Saudi Arabia; 33 Obstetrics/Gynecology Department, King Faisal Specialist Hospital and Research Centre, Riyadh, 11211 Kingdom of Saudi Arabia; 34 Department of Cell Biology, King Faisal Specialist Hospital and Research Centre, Riyadh, 11211 Kingdom of Saudi Arabia; 35 Department of Biostatistics, Epidemiology and Scientific Computing, King Faisal Specialist Hospital and Research Centre, Riyadh, 11211 Kingdom of Saudi Arabia; 36 Medical Genetics Research Center, Shahid Sadoughi University of Medical Sciences, Yazd, Iran; 37 Yazd Reproductive Sciences Institute, Shahid Sadoughi University of Medical Sciences, Yazd, Iran; 38 Diabetes Research Center, Shahid Sadoughi University of Medical Sciences, Yazd, Iran; 39 Department of Neurosurgery, Program On Neurogenetics, Yale School of Medicine, Yale University, New Haven, CT USA; 40 Masonic Medical Research Institute, Utica, NY USA; 41 Department of Pediatrics, Pediatric Neurology Clinic, Choithram Hospital and Research Centre, Indore, Madhya Pradesh India; 42 Pediatric Gastroenterology Department, Children’s Hospital and Institute of Child Health, Lahore, Pakistan

**Keywords:** Epileptic encephalopathy, UGP2, ATG mutations, Start-loss mutation, Genetics, Whole exome sequencing, Microcephaly, Recurrent mutation, Founder mutation, Essential gene

## Abstract

Developmental and/or epileptic encephalopathies (DEEs) are a group of devastating genetic disorders, resulting in early-onset, therapy-resistant seizures and developmental delay. Here we report on 22 individuals from 15 families presenting with a severe form of intractable epilepsy, severe developmental delay, progressive microcephaly, visual disturbance and similar minor dysmorphisms. Whole exome sequencing identified a recurrent, homozygous variant (chr2:64083454A > G) in the essential *UDP*-*glucose pyrophosphorylase* (*UGP2*) gene in all probands. This rare variant results in a tolerable Met12Val missense change of the longer UGP2 protein isoform but causes a disruption of the start codon of the shorter isoform, which is predominant in brain. We show that the absence of the shorter isoform leads to a reduction of functional UGP2 enzyme in neural stem cells, leading to altered glycogen metabolism, upregulated unfolded protein response and premature neuronal differentiation, as modeled during pluripotent stem cell differentiation in vitro. In contrast, the complete lack of all UGP2 isoforms leads to differentiation defects in multiple lineages in human cells. Reduced expression of Ugp2a/Ugp2b in vivo in zebrafish mimics visual disturbance and mutant animals show a behavioral phenotype. Our study identifies a recurrent start codon mutation in *UGP2* as a cause of a novel autosomal recessive DEE syndrome. Importantly, it also shows that isoform-specific start-loss mutations causing expression loss of a tissue-relevant isoform of an essential protein can cause a genetic disease, even when an organism-wide protein absence is incompatible with life. We provide additional examples where a similar disease mechanism applies.

**Electronic supplementary material:**

The online version of this article (10.1007/s00401-019-02109-6) contains supplementary material, which is available to authorized users.

## Introduction

Developmental and/or epileptic encephalopathies (DEEs) are a heterogeneous group of genetic disorders, characterized by severe epileptic seizures in combination with developmental delay or regression [49]. Genes involved in multiple pathophysiological pathways have been implicated in DEEs, including synaptic impairment, ion channel alterations, transporter defects and metabolic processes such as disorders of glycosylation [68]. Mostly, dominant acting, de novo mutations have been identified in children suffering from DEEs [32], and only a limited number of genes with a recessive mode of inheritance are known so far, with a higher occurrence rate in consanguineous populations [71]. A recent cohort study on DEEs employing whole exome sequencing (WES) and copy number analysis, however, found that up to 38% of diagnosed cases might be caused by recessive genes, indicating that the importance of this mode of inheritance in DEEs has been underestimated [74].

The human genome contains ~ 20,000 genes of which more than 5000 have been implicated in genetic disorders. Wide-scale population genomic studies and CRISPR–Cas9-based loss-of-function (LoF) screens have identified around 3000–7000 genes that are essential for the viability of the human organism or result in profound loss of fitness when mutated, in agreement with that they are depleted for LoF variants in the human population [10]. For some of these essential genes, it is believed that LoF variants are incompatible with life and are, therefore, unlikely to be implicated in genetic disorders presenting in postnatal life [84]. One such example is the *UDP*-*glucose pyrophosphorylase* (*UGP2*) gene at chromosome 2. UGP2 is an essential octameric enzyme in nucleotide sugar metabolism [38, 39, 121], as it is the only known enzyme capable of catalyzing the conversion of glucose-1-phosphate to UDP-glucose [36, 108]. UDP-glucose is a crucial precursor for the production of glycogen by *glycogen synthase* (GYS) [2, 44], and also serves as a substrate for *UDP*-*glucose*:*glycoprotein transferases* (UGGT) and *UDP*-*glucose*-*6*-*dehydrogenase* (UGDH), thereby playing important roles in glycoprotein folding control, glycoconjugation and UDP-glucuronic acid synthesis. The latter is an obligate precursor for the synthesis of glycosaminoglycans and proteoglycans of the extracellular matrix [65, 110], of which aberrations have been associated with DEEs and neurological disorders [4, 24, 77, 98]. *UGP2* has previously been identified as a marker protein in various types of malignancies including gliomas where its upregulation is correlated with a poor disease outcome [27, 59, 61, 101, 103, 111, 112, 122], but has so far not been implicated in genetic diseases and it has been speculated that this is given its essential role in metabolism [38].

Many genes are differentially expressed amongst tissues, regulated by non-coding regulatory elements [76]. In addition, it has become clear that there are more than 40,000 protein isoforms encoded in the human genome, whose expression levels vary amongst tissues. Although there are examples of genetic disorders caused by the loss of tissue-specific protein isoforms [41, 47, 57, 100], it is unknown whether a tissue-relevant loss of an essential gene can be involved in human disease. Here, we report on such a scenario, providing evidence that a novel form of a severe DEE syndrome is caused by the brain-relevant loss of the essential gene *UGP2* due to an isoform-specific and germ line-transmitted start codon mutation. We present data that this is likely a more frequent disease mechanism in human genetics, illustrating that essential genes for which organism-wide loss is lethal can still be implicated in genetic disease when only absent in certain tissues due to expression misregulation.

## Methods

### Patient recruitment

All affected probands were investigated by their referring physicians and all genetic analyses were performed in a diagnostic setting. Legal guardians of affected probands gave informed consent for genomic investigations and publication of their anonymized data.

### Next-generation sequencing of index patients

#### Individual 1

Genomic DNA was isolated from peripheral blood leukocytes of the proband and both parents, and exome-coding DNA was captured with the Agilent SureSelect Clinical Research Exome (CRE) kit (v2). Sequencing was performed on an Illumina HiSeq 4000 with 150-bp paired-end reads. Reads were aligned to hg19 using BWA (BWA-MEM v0.7.13) and variants were called using the GATK haplotype caller (v3.7 (reference: https://www.broadinstitute.org/gatk/) [67]. Detected variants were annotated, filtered and prioritized using the Bench lab NGS v5.0.2 platform (Agilent technologies). Initially, only genes known to be involved in epilepsy were analyzed, followed by a full exome analysis revealing the homozygous *UGP2* variant.

#### Individuals 2, 3 and 4

Using genomic DNA from the proband and parents (individual 4) or the proband, parents, and affected sibling (individuals 2 and 3), the exonic regions and flanking splice junctions of the genome were captured using the SureSelect Human All Exon V4 (50 Mb) (individual 4) or the IDT xGen Exome Research Panel v1.0 (individuals 2 and 3). Massively parallel (NextGen) sequencing was done on an Illumina system with 100 bp or greater paired-end reads. Reads were aligned to human genome build GRCh37/UCSC hg19 and analyzed for sequence variants using a custom-developed analysis tool. Additional sequencing technology and variant interpretation protocol has been previously described [82]. The general assertion criteria for variant classification are publicly available on the GeneDx ClinVar submission page (https://www.ncbi.nlm.nih.gov/clinvar/submitters/26957/).

#### Individual 5

Diagnostic exome sequencing was done at the Departments of Human Genetics of the Radboud University Medical Center Nijmegen, The Netherlands, and performed essentially as described previously [96].

#### Individuals 6, 7, 8, 9, 10, 14, 15, 16, 17 and 18

After informed consent, we collected blood samples from the probands, their parents and unaffected siblings, and extracted DNA using standard procedures. To investigate the genetic cause of the disease, WES was performed in the affected proband. Nextera Rapid Capture Enrichment kit (Illumina) was used according to the manufacturer’s instructions. Libraries were sequenced in an Illumina HiSeq3000 using a 100-bp paired-end reads protocol. Sequence alignment to the human reference genome (UCSC hg19), variants calling, and annotation were performed as described elsewhere [69]. After removing all synonymous changes, we filtered single nucleotide variants (SNVs) and indels, only considering exonic and donor/acceptor splicing variants. In accordance with the pedigree and phenotype, priority was given to rare variants [< 1% in public databases, including 1000 Genomes project, NHLBI Exome Variant Server, Complete Genomics 69, and Exome Aggregation Consortium (ExAC v0.2)] that were fitting a recessive or a de novo model. After identifying the *UGP2* variant in the proband, Sanger sequencing was used to confirm segregation in other affected and unaffected family members.

#### Individuals 11, 20, 21 and 22

Whole exome sequencing was performed at CENTOGENE AG, as previously described [105].

#### Individuals 12 and 13

High-quality DNA was used to capture exons using the SureSelect kit (Agilent, Santa Clara, CA, US). Then genomic libraries were created according to the manufacturer’s protocols. Sequences were read on Proton (Life Technologies Inc., Carlsbad, CA, US). Downstream analyses such as sequence alignment, indexing and raw variant calling were done using publicly and commercially available tools such as Ion Reporter, SAMTools, and Genomic Analysis ToolKit. Moreover, variant interrogations were done using sequence-variant databases, such as dbSNP, Ensembl, and the National Heart, Lung, and Blood Institute (NHLBI) Exome Variant Server (EVS), 1000 genome project.

#### Individual 19

Whole exome sequencing was performed in a diagnostic setting at MEDGENOME, India. DNA extracted from blood was used to perform targeted gene capture using the Agilent SureSelect V5 exome capture kit. The libraries were sequenced to mean > 80–100 × coverage on Illumina sequencing platform. GATK best practices framework was used for variant identification using Sentieon (v201808.01), sequences obtained were aligned to GRCh37/hg19 using Sentieon aligner and analyzed using Sentieon for removing duplicates, recalibration and re-alignment of indels. Sentieon haplotypecaller has been used to identify variants which are relevant to the clinical indication. Gene annotation of the variants was performed using VEP program against the Ensemble release 91 human gene model.

### Human brain samples

Tissue was obtained, upon informed consent, and used in a manner compliant with the Declaration of Helsinki and the Research Code provided by the local ethical committees. Fetal brains were preserved after spontaneous or induced abortions with appropriate written consent for brain autopsy and use of rest material for research. We performed a careful histological and immunohistochemical analysis, and evaluation of clinical data (including genetic data, when available). We only included specimens displaying a normal cortical structure for the corresponding age and without any significant brain pathology.

### Brain tissue immunohistochemistry

For immunohistochemical analysis, we used two cases from the first trimester (GW6 and GW9), four cases from the second trimester (GW21, GW23, GW24 and GW26) and two cases from the third trimester (GW33 and GW36). Anatomical regions were determined according to the atlas of human brain development [11–14]. We cut 4-µm sections from formalin-fixed, paraffin-embedded whole fetuses (GW6 and GW9) and brain tissue from cerebral, mesencephalic, cerebellar and brain stem regions (from GW21 to GW36). Slides were stained with mouse anti-UGP2 (C-6) in a 1:150 dilution (Santa Cruz) and visualized using Mouse and Rabbit Specific HRP/DAB (ABC) Detection IHC kit (Abcam). Mayer’s hematoxylin was used as a counterstain for immunohistochemistry followed by mounting and coverslipping (Bio-Optica) for slides. Prepared slides were analyzed and scanned under a VisionTek^®^ Live Digital Microscope (Sakura).

### Cloning of UGP2 cDNA

RNA was isolated using TRI reagent (Sigma) from whole peripheral blood of index patient 1 and her parents, after red blood cell depletion with RBC lysis buffer (168 mM NH_4_Cl, 10 mM KHCO_3_, 0.1 mM EDTA). cDNA was synthesized following the iSCRIPT cDNA Synthesis Kit (Bio-Rad) protocol, and the coding sequence of the long and short UGP2 isoform (wild type or mutant) was PCR amplified together with homology arms for Gibson assembly (see Supplementary Table 8, online resource, for primer sequences) using Phusion High-Fidelity DNA polymerase (NEB). PCR-amplified DNA was then cloned by Gibson assembly as previously described [9] in a pPyCAG-IRES-puro plasmid (a kind gift from Ian Chambers, Edinburgh) opened with EcoRI for experiments in mammalian cells. All obtained plasmids were sequence verified by Sanger sequencing (complete plasmid sequences available upon request).

### Fibroblast cell culture

Fibroblasts from index patient 1 and her parents were obtained using a punch biopsy according to standard procedures, upon informed consent (IRB approval MEC-2017-341). Fibroblasts from the parents of index patients 2 and 3 were also obtained upon informed consent at McMaster Children’s Hospital. All fibroblasts were cultured in standard DMEM medium supplemented with 15% fetal calf serum, MEM non-essential amino acids (Sigma), 100 U/ml penicillin and 100 µg/ml streptomycin, as done previously [6], in routine humidified cell culture incubators at 20% O_2_. Fibroblast cell lines were transfected using Lipofectamine 3000 (Invitrogen) with the indicated plasmid constructs. All cell lines used in this report were regularly checked for the presence of mycoplasma and were negative during all experiments.

### Genome engineering in human embryonic stem cells

H9 human embryonic stem cells were cultured as previously described [8, 9]. In short, cells were maintained on feeder-free conditions in mTeSR-1 medium (STEMCELL technologies) on Matrigel (Corning)-coated culture dishes. To engineer the patient-specific *UGP2* mutation by homologous recombination [7], ESCs were transfected using Lipofectamine 3000 with a plasmid expressing eSpCas9-t2a-GFP (a kind gift of Feng Zhang) and a gRNA targeting the *UGP2* gene (see Supplementary Table 8, online resource, for the sequence), together with a 60-bp single-stranded oligonucleotide (ssODN) homology template encoding the patient mutation (synthesized at IDT). To increase the stability of the ssODN and, therefore, homologous recombination efficiency, the first two 5′ and 3′ nucleotides were synthesized using phosphorothiorate bonds [80]. 48 h post-transfection, GFP-expressing cells were sorted, and 6000 single GFP-positive cells were plated on a Matrigel-coated six-well plate in the presence of 10 µM ROCK-inhibitor (Y27632, Millipore). After approximately 10 days, single colonies where manually picked, expanded and genotyped using Sanger sequencing (see Supplementary Table 8, online resource, for primer sequences). As a by-product of non-homologous end joining, knockout clones were obtained which showed a single nucleotide A insertion at position 42 of *UGP2* transcript 1 (chr2:64083462_64083463insA), leading to an out-of-frame transcript and a premature termination of the protein at amino acid position 47 (D15Rfs*33). Western blotting confirmed the absence of all UGP2 proteins in knockout clones and the loss of the short UGP2 isoform in clones with the patient mutation. To produce a stable rescue cell line, ESCs were transfected as previously described with the pPyCAG-IRES-puro plasmid expressing either the long WT or mutant UGP2 isoform. After 48 h, the population of cells with the transgene integration was selected with 1 µg/ml puromycin. Engineered ESC clones had a normal colony morphology and pluripotency factor expression.

### Patient-specific induced pluripotent stem cell generation

Patient fibroblast cell lines were reprogrammed using the CytoTune™-iPS 2.0 Sendai Reprogramming Kit (Thermo Scientific, A16517) expressing the reprogramming factors OCT4, SOX2, KLF4 and C-MYC on Matrigel-coated cell culture plates, upon informed consent (IRB approval MEC-2017–341). After approximately 4–5 weeks, emerging colonies were manually picked and expanded. Multiple clones were assessed for their karyotype, pluripotency factor expression and three lineage differentiation potential (Stem Cell Technologies, #05230), following the routine procedures of the Erasmus MC iPS Cell core facility, as previously described [6]. Sanger sequencing was used to verify the genotype of each obtained iPSC line. We used three validated clones for each individual in our experiments.

### Neural stem cell differentiation

Pluripotent cells were differentiated in neural stem cells (NSCs), using a modified dual SMAD inhibition protocol [20]. In short, 18,000 cells/cm^2^ were plated on Matrigel-coated cell culture dishes in mTeSR-1 medium in the presence of 10 µM Y27632. When cells reached 90% confluency, the medium was switched to differentiation medium (KnockOut DMEM (Gibco), 15% KnockOut serum replacement (Gibco), 2 mM l-glutamine (Gibco), MEM non-essential amino acids (Sigma), 0.1 mM β-mercaptoethanol, 100U/ml penicillin and 100 µg/ml streptomycin) supplemented with 2 µM A 83-01 (Tocris) and 2 µM Dorsomorphin (Sigma-Aldrich). At day 6, medium was changed to an equal ratio of differentiation medium and NSC medium (KnockOut DMEM-F12 (Gibco), 2 mM l-glutamine (Gibco), 20 ng/ml bFGF (Peprotech), 20 ng/ml EGF (Peprotech), 2% StemPro Neural supplement (Gibco), 100U/ml penicillin and 100 µg/ml streptomycin) supplemented with 2 µM A 83-01 (Tocris) and 2 µM Dorsomorphin (Sigma-Aldrich). At day 10, cells were passaged (NSC *p* = 0) using Accutase (Sigma) and maintained in NSC medium. We used commercially available H9-derived NSCs (Gibco) as a control (a kind gift from Raymond Poot, Rotterdam).

### Other stem cell differentiation experiments

ESCs were differentiated into hematopoietic stem cells and cardiomyocytes using commercially available STEMCELL technology kits (STEMdiff Hematopoietic kit #05310, STEMdiff Cardiomyocyte differentiation kit #05010) according to the manufacturer’s instructions. Cells were finally harvested and lysed with TRI reagent to isolate RNA for further qRT-PCR analysis.

### RNA-sequencing and data analysis

For patient RNA-seq, peripheral blood was obtained from index patient 1 and her parents, collected in PAX tubes and RNA was isolated following standard diagnostic procedures in the diagnostics unit of the Erasmus MC Clinical Genetics department. RNA-seq occurred in a diagnostic setting, and sequencing was performed at GenomeScan (Leiden, The Netherlands). For RNA-seq of in vitro-cultured cell lines, RNA was obtained from six-well cultures using TRI reagent, and further purified using column purification (Qiagen, #74204). mRNA capture, library prep including barcoding and sequencing on an Illumina HiSeq2500 machine were performed according to standard procedures of the Erasmus MC Biomics facility. Approximately 20 million reads were obtained per sample. For cell line experiments, two independent H9 wild-type cultures, two independent knockout clones harboring the same homozygous *UGP2* genetic alteration and two independent clones harboring the patient homozygous *UGP2* mutation were used. Each cell line was sequenced in two technical replicates at ESC state and differentiated NSC state (at passage 5). FASTQ files obtained after de-multiplexing of single-end, 50-bp sequencing reads were trimmed by removing possible adapters using Cutadapt after quality control checks on raw data using the FastQC tool. Trimmed reads were aligned to the human genome (hg38) using the HISAT2 aligner [50]. To produce Genome Browser Tracks, aligned reads were converted to bedgraph using bedtools genomecov, after which the bedGraphToBigWig tool from the UCSC Genome Browser was used to create a bigwig file. Aligned reads were counted for each gene using htseq-count [3] and GenomicFeatures [55] was used to determine the gene length by merging all non-overlapping exons per gene from the Homo_sapiens.GRCh38.92.gtf file (Ensemble). Differential gene expression and RPKM (Reads Per Kilobase per Million) values were calculated using edgeR [85] after removing low-expressed genes and normalizing data. The threshold for significant differences in gene expression was FDR < 0.05. To obtain a list of ESC and NSC reference genes used in Supplementary Fig. 6F, online resource, we retrieved genes annotated in the following GO terms using GSEA/MSigDB web site v7.0: GO_FOREBRAIN_NEURON_DEVELOPMENT (GO:0021884), GO_CEREBRAL_CORTEX_DEVELOPMENT (GO:0021987), GO_NEURAL_TUBE_DEVELOPMENT (GO:0021915), BHATTACHARYA_EMBRYONIC_STEM_CELL (PMID: 15070671) and BENPORATH_NOS_TARGETS (PMID: 18443585).

### Functional enrichment analysis

Metascape [123], g:profiler [79] and Enrichr [52] were used to assess functional enrichment of differentially expressed genes. Supplementary Table 4, online resource, reports all outputs in Log*P*, log(*q* value) and Adjusted *p* value (*q* value) for Metascape and g:profiler, and in *p* value, Adjusted *p* value (*q* value) and combined score (which is the estimation of significance based on the combination of Fisher's exact test *p* value and *z* score deviation from the expected rank) for Enrichr. All tools were used with default parameters and whole genome set as background.

### Genome-wide homology search

To make a genome-wide list of transcripts sharing a similar structure as *UGP2* transcripts, 42,976 transcripts from 21,522 genes (Human genes GRCh38.p12) were extracted using BioMart of Ensembl (biomaRt R package). 11,056 out of 21,522 genes had only 1 transcript and the remaining 31,920 transcripts from 10,466 genes were selected, the protein sequences were obtained with biomaRt R package and homology analysis was performed using the NCBI’s blastp (formatting option: -outfmt = 6) command line. We grouped longest and shorter transcript based on coding sequence length and only kept those that matched a pairwise homology comparison between the longest and the shorter transcript with the following criteria: complete 100 percent identity, without any gap and mismatch, and starting ATG codon of shortest transcript being part of the longest transcript(s). 1766 genes meet these criteria. We then filtered these genes for published essential genes [10], leaving us with 1197 genes. Using BioMart (Attributes: Phenotype description and Study external reference) of Ensembl we then evaluated the probability that these genes were implicated in disease and identified 850 genes that did not have an association with disease phenotype/OMIM number. Of those, 247 genes encoded proteins of which the shorter isoform differed less than 50 amino acids from the longer isoform. We chose this arbitrary threshold to exclude those genes where both isoforms could encode proteins differing largely in size and might, therefore, encode functionally completely differing proteins (although we cannot exclude that this will also hold true for some of the genes in our selection).

### Differential isoform expression in fetal tissues

Publically available RNA-seq data from various fetal tissue samples (Supplementary Table 2, online resource) were analyzed using the same workflow as described for the RNA-seq data analysis above. To determine differential isoform expression in these tissues, we calculated a ratio between the unique exon(s) of the shortest and longest transcript for each gene and assessed its variability across different fetal tissue samples. The number of reads for each unique exon of a transcript was calculated by mapping aligned RNA-seq reads against the unique exon coordinate using bedtools multicov. The longest and shortest transcripts were separated and the transcript ratio (number of counts of shortest transcript/(number of counts of shortest transcript + number of counts of longest transcript)) for each gene was obtained from the average reads of RNA-seq samples per tissue. 382 genes out of 1197 genes showed high variability across different samples (defined as a difference between highest and lowest ratio > 0.5), 277 of those highly variable genes were not associated with a disease phenotype/OMIM number and of these 83 genes had a length less than 50 amino acids (a subset of the 247 genes with no OMIM and length less than 50 amino acids).

### Haplotype analysis

The 30 MB region surrounding *UGP2* was extracted from exome sequencing VCF files to include both common and rare polymorphisms. Variants were filtered for a minimum depth of coverage of at least 10 reads and a genotype quality of at least 50. The filtered variants were then used as input in PLINK (v1.07) with the following settings:

- homozyg-snp 5
- homozyg-kb 100
- homozyg-gap 10,000
- homozyg-window-het 0

ROH around the *UGP2* variant was identified in all five probands examined. The minimum ROH in common between all samples was a 5-Mb region at chr2: 60679942–65667235. We note that targeted sequencing leads to uneven SNP density, so the shared ROH may, in fact, be larger or smaller. Next, we used recombination maps from deCODE to estimate the size of the region in centiMorgans (cM). We then used the region size in cM to estimate the time to event in generations using methods previously described [120].

### qPCR analysis

RNA was obtained using TRI reagent, and cDNA prepared using iSCRIPT cDNA Synthesis Kit according to the manufacturer’s instructions. qPCR was performed using iTaq universal SYBR Green Supermix in a CFX96RTS thermal cycler (Bio-Rad). Supplementary Table 8, online resource, summarizes all primers used in this study. Relative gene expression was determined following the ΔΔct method. To calculate the ratio of the short isoform, we performed absolute quantification as previously described [109]. Briefly, we performed qPCR on known copy numbers, ranging from 10^3^ to 10^8^ copies, of a plasmid containing the short *UGP2* isoform (5′ UTR included) using primers detecting specifically either the total or the short isoform. After plotting the log copy number versus the ct, we obtained a standard curve that we used to extrapolate the copy number of the unknown samples. To test for significance, we used Student’s *T* test and considered *p* < 0.05 as significant.

### Western blotting

Proteins were extracted with NE buffer (20 mM HEPES, pH 7.6, 1.5 mM MgCl_2_, 350 mM KCl, 0.2 mM EDTA and 20% glycerol) supplemented with 0.5% NP40, 0.5 mM DTT, cOmplete Protease Inhibitor Cocktail (Roche) and 150 U/ml benzonase. Protein concentration was determined by BCA (Pierce) and 20–50 µg of proteins was loaded onto a 4–15% Criterion TGX gel (Bio-Rad). Proteins were then transferred to a nitrocellulose membrane using the Trans-Blot Turbo Transfer System (Bio-Rad). The membrane was blocked in 5% milk in PBST and subsequently incubated overnight at 4 °C with primary antibody diluted in milk. After PBST washes, the membrane was incubated 1 h at RT with the secondary antibody and imaged with an Odyssey CLX scanning system (Li-Cor). Band intensities were quantified using Image Studio (Li-cor). Antibodies used were Ms-α-UGP2 (sc-514174) 1:250; Ms-α-Vinculin (sc-59803) 1:10,000; Gt-α-actin (sc-1616) 1:500; Ms-α-LAMP2 (H4B4) 1:200; IRDye 800CW Goat anti-Mouse (926-32210) 1:5000; IRDye 680 Donkey anti-Goat (926-32224) 1:5000.

### Zebrafish disease modeling

Animal experiments were approved by the Animal Experimentation Committee at Erasmus MC, Rotterdam. Zebrafish embryos and larvae were kept at 28 °C on a 14–10‐h light–dark cycle in 1 M HEPES buffered (pH 7.2) E3 medium (34.8 g NaCl, 1.6 g KCl, 5.8 g CaCl_2_·2H_2_O, 9.78 g MgCl_2_·6H_2_O). For live imaging, the medium was changed at 1 dpf to E3 + 0.003% 1‐phenyl 2‐thiourea (PTU) to prevent pigmentation. *Ugp2a* and *ugp2b* were targeted by Cas9/gRNA RNP complex as we did before [51]. Briefly, fertilized oocytes from a tgBAC(slc1a2b:Citrine)re01tg reporter line [51] maintained on an TL background strain were obtained, and injected with Cas9 protein and crRNA and tracrRNA synthesized by IDT (Alt-R CRISPR–Cas9 System), targeting the open reading frame of zebrafish *ugp2a* and *ugp2b*. DNA was extracted from fin clips and used for genotyping using primers flanking the gRNA location (Supplementary Table 8, online resource) followed by sequencing. Mutants with a high level of out-of-frame indels in both genes were identified using TIDE [18] and intercrossed to obtain germ line transmission. Upon re-genotyping, mutant zebrafish with the following mutations as indicated in Fig. 6 were selected and further intercrossed. In this study, we describe two new mutant fish lines containing deletions in *ugp2a* (*ugp2a*^Δ/Δ^) and ugp2b (*ugp2b*^Δ/Δ^): *ugp2a*^*re08/re08*^ containing a 37 bp deletion in exon 2 and *ugp2b*^*re09/re09*^ containing a 5 bp deletion in exon 2. Intravital imaging, and analysis of eye movement, was performed as previously described [51]. Briefly, zebrafish larvae anesthetized in tricaine were mounted in low-melting point agarose-containing tricaine and imaged using a Leica SP5 intravital imaging setup with a 20 × /1.0 NA water-dipping lens. To assess the locomotor activity of zebrafish larvae from 3 to 5 dpf, locomotor activity assays were performed using an infrared camera system (DanioVision™ Observation chamber, Noldus) and using EthoVision^®^ XT software (Noldus) as described [51]. Briefly, control (*n* = 24) and *ugp2a*^Δ/Δ^; *ugp2b*^Δ/Δ^ (*n* = 24) zebrafish larvae, in 48-well plates, were subjected to gradually increasing (to bright light) and decreasing light conditions (darkness) as in Kuil et al. [51]. Distance traveled (mm) per second was measured. For 4-AP (Sigma) stimulation, animals were treated with 4-AP dissolved in DMSO 30 min before the onset of the experiments. For these experiments, locomotor activity was measured over 35 min, with the first 5 min going from dark to light, followed by 30 min under constant light exposure.

### Periodic acid–Schiff (PAS) staining

ESCs or differentiated NSCs (wild type, KO, KI or rescue) were incubated under hypoxia conditions (3% O_2_) for 48 h. Cells were fixed with 5.2% formaldehyde in ethanol, incubated 10 min with 1% periodic acid, 15 min at 37 °C with Schiff’s reagent (Merck) and 5 min with hematoxylin solution (Klinipath) prior to air drying and mounting. Every step of the protocol is followed by a 10-min wash with tap water. Imaging occurred on an Olympus BX40 microscope. Images were acquired at a 100 × magnification, and ImageJ software was used for quantification. For ESCs, we used a minimum of 20 images per genotype for the quantification, containing on average 20 cells each, calculating the percentage of PAS-positive area. For NSCs, we imaged between 80 and 100 cells per genotype, counting the number of glycogen granules in the cytoplasm. We report the average of two independent experiments at 48 h low oxygen.

### UGP2 enzymatic activity

The measurement of UGP2 enzyme activity was performed according to a modified GALT enzyme activity assay as described previously [62]. Frozen cell pellets were defrosted and homogenized on ice. 10 µl of each cell homogenate (around 0.5 mg protein/ml as established by BSA protein concentration determination) was pre-incubated with 10 µl of dithiothreitol (DDT) for 5 min at 25 °C. 80 µl of a mixture of glucose-1-phosphate (final concentration 1 mM), UTP (0.2 mM), magnesium chloride (1 mM), glycine (125 mM) and Tris–HCl (pH8) (40 mM) was added and incubated for another 15 min at 25 °C. The reaction was stopped by adding 150 µl of 3.3% perchloric acid. After 10 min on ice, the mixture was centrifuged (10,000 rpm for 5 min at 4 °C), the supernatant isolated and neutralized with ice-cold 8 µl potassium carbonate for 10 min on ice. After centrifugation, the supernatant was isolated and 1:1 diluted with eluent B (see below) after which the mixture was added to a MilliPore Amicon centrifugal filter unit. After centrifugation, the supernatant was stored at − 20 °C until use. The separation was performed by injection of 10 µl of the defrosted supernatant onto a HPLC system with UV/VIS detector (wavelength 262 nm) equipped with a reversed-phase Supelcosil LC-18-S 150 mm × 4.6 mm, particle size 5 µm, analytical column and Supelguard LC18S guard column (Sigma-Aldrich). During the experiments, the temperature of the column was maintained at 25 °C. The mobile phase consisted of eluent A (100% methanol) and eluent B (50 mM ammonium phosphate buffer pH 7.0 and 4 mM tetrabutylammonium bisulphate). A gradient of 99% eluent B (0–20 min), 75% eluent B (20–30 min) and 99% eluent B (30–45 min) at a flow rate of 0.5 m/min was used. The reaction product UDP-glucose was quantified using a calibration curve with known concentrations of UDP-glucose. UGP2 activity was expressed as the amount of UDP-glucose formed per mg protein per min. Experiments were performed in duplicate and for every cell line two independently grown cell pellets were used.

### Immunostaining/immunohistochemistry

For immunofluorescence staining, cells were seeded on coverslips coated with 100 µg/ml poly-d-lysine (Sigma) overnight. For ESC, coverslips were further coated with Matrigel (Corning) for 1 h at 37 °C. At 70% confluency, cells were fixed with 4% PFA for 15 min at RT. Cells were then permeabilized with 0.5% Triton in PBS, incubated 1 h in blocking solution (3% BSA in PBS) and then overnight at 4 °C with the primary antibody diluted in blocking solution. The next day coverslips were incubated 1 h at room temperature in the dark with a Cy3-conjugated secondary antibody and mounted using ProLong Gold antifade reagent with DAPI (Invitrogen) to counterstain the nuclei. Images were acquired with a ZEISS Axio Imager M2 using a 63X objective.

## Data availability

RNA-Seq of in vitro studies is publicly available through the National Center for Biotechnology Information (NCBI) Gene Expression Omnibus (GEO) under accession number GSE137129. Due to privacy regulations and consent, raw RNA-seq data from patient blood and genomic sequencing data cannot be made available. To retrieve tissue wide expression levels of *UGP2*, the GTEx Portal was accessed on 16/07/2019 (https://gtexportal.org/home/). RNA-seq data from various tissues were downloaded from various publications [46, 83, 94, 118]. All publically available data that were re-analyzed here are summarized in Supplementary Table 2, online resource.

## Results

### A recurrent ATG mutation in UGP2 in 22 individuals presenting with a severe DEE

**Fig. 1:**
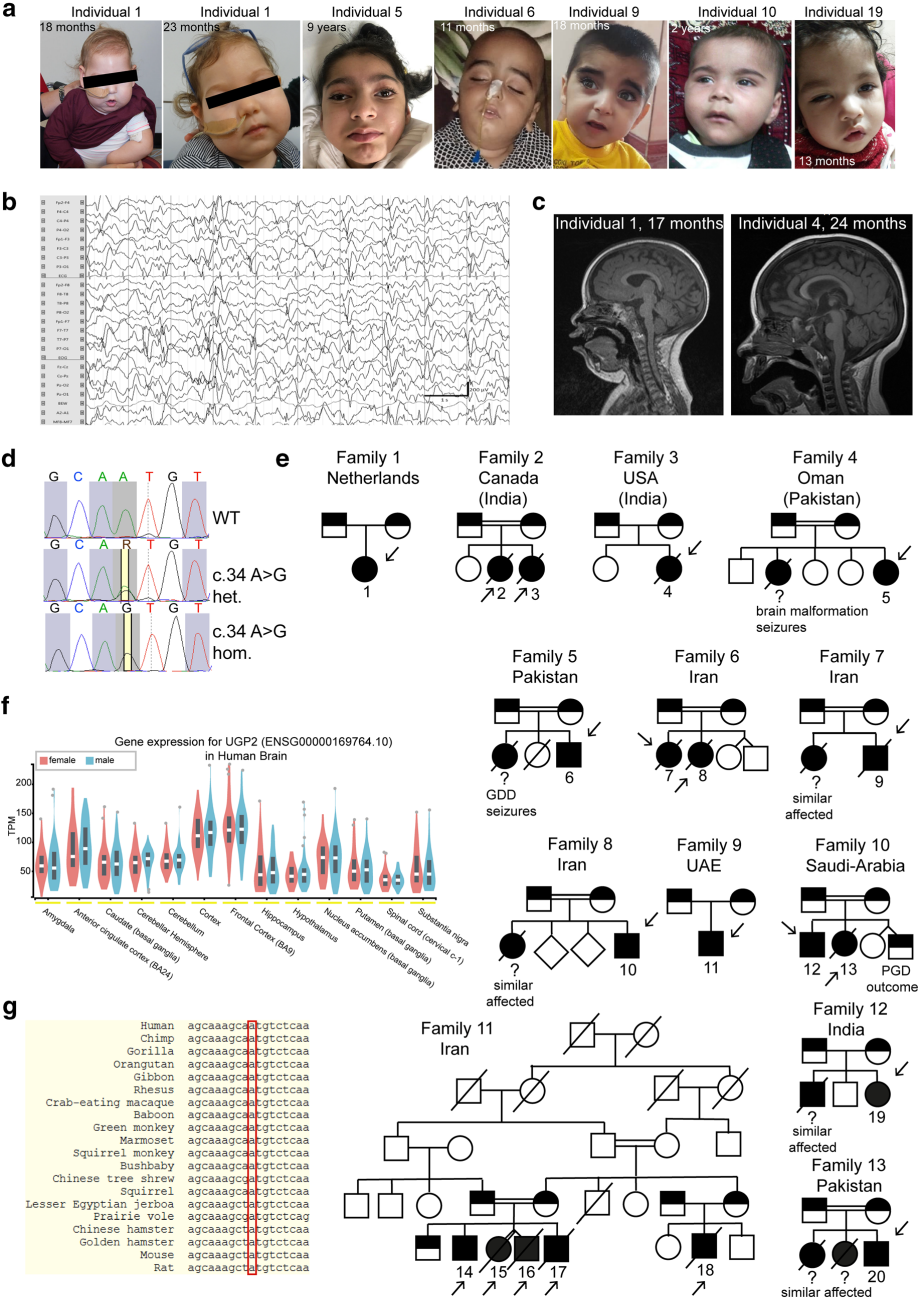
UGP2 homozygous variants in 20 individuals with severe epileptic encephalopathy. **a** Facial pictures of individual 1 (at 18 and 23 months), individual 5 (at 9 years), individual 6 (at 11 months), individual 9 (at 18 months), individual 10 (at 2 years) and individual 19 (at 13 months). Note the progressive microcephaly with sloping forehead, suture ridging, bitemporal narrowing, high hairline, arched eyebrows, pronounced philtrum, a relatively small mouth and large ears. **b** Electroencephalogram of individual 1 at the age of 8 months showing a highly disorganized pattern with high-voltage irregular slow waves intermixed with multifocal spikes and polyspikes. **c** T1-weighted mid-sagittal brain MRI of individual 1 (at 17 months) and individual 4 (at 24 months) illustrating global atrophy and microcephaly but no major structural anomalies. **d** Sanger sequencing traces of family 1, confirming the chr2:64083454A>G variant in *UGP2* in heterozygous and homozygous states in parents and affected individual 1, respectively. **e** Family pedigrees of ascertained patients. Affected individuals and heterozygous parents are indicated in black and half black, respectively. Affected individuals with confirmed genotype are indicated with an arrow, and numbers. Other not-tested  affected siblings presenting with similar phenotypes are indicated with a question mark. Consanguineous parents are indicated with a double connection line. Males are squares, females are circles; unknown sex is indicated with rotated squares; deceased individuals are marked with a line. **f** Violin plots showing distribution of gene expression (in TPM) amongst male and female samples from the GTEx portal for various brain regions. Outliers are indicated by dots. **g** Multiple species sequence alignment from the UCSC browser, showing that the ATG start site is highly conserved

We encountered a 3-month-old girl (Fig. 1a, family 1, individual 1) that was born as the first child to healthy non-consanguineous Dutch parents, by normal vaginal delivery after an uneventful pregnancy conceived by ICSI. She presented in the first weeks of life with irritability and jitteriness, which developed into infantile spasms and severe epileptic activity on multiple electroencephalograms, giving rise to a clinical diagnosis of West syndrome (Fig. 1b). Despite the use of multiple anti-epileptic drugs, including ACTH and a ketogenic diet, seizures remained intractable and occurred daily. Severe developmental delay was evident without acquisition of any noticeable developmental milestones, causing the need for gastrointestinal tube feeding. Visual tracking was absent, and foveal hypopigmentation, hypermetropia and mild nystagmus were noticed upon ophthalmological investigation. MRI brain imaging showed no gross structural abnormalities or migration disorders at the age of 4 months, but displayed reduced white matter, that further developed into global atrophy with wide sulci and wide pericerebral liquor spaces at the age of 17 months (Fig. 1c, Supplementary Fig. 1b, online resource). At that time, she had become progressively microcephalic, with a head circumference of − 2.96 SD at the last investigation at 23 months of age (Supplementary Fig. 1a, online resource). She showed a number of minor dysmorphisms, including a sloping forehead, elongated head with suture ridging, bitemporal narrowing, a relatively small mouth and large ears (Fig. 1a). Neurological examination showed brisk, symmetric deep tendon reflexes, more pronounced at the upper limbs. Routine investigations, including metabolic screening in urine, plasma and cerebrospinal fluid were normal. A SNP-array showed a normal female chromosomal profile, with a large, ~ 30 Mb run of homozygosity (ROH) at chromosome 2, and a few smaller ROH regions, adding up to 50 Mb ROH regions in total, pointing to an unrecognized common ancestor of both parents (coefficient of inbreeding 1/64). Subsequent trio WES did not show any disease-causing variants in known DEE genes, but identified a homozygous variant (chr2:64083454A>G) in *UGP2*, located in the large ROH region (Fig. 1d), with no other disease-implicated variants observed in that region. Both parents were heterozygous carriers of the same variant. Via Genematcher [97] and our network of collaborators, we identified 21 additional individuals from 14 unrelated families (of which 10 were consanguineous), harboring the exact same homozygous variant and presenting with an almost identical clinical phenotype of intractable seizures, severe developmental delay, visual disturbance, microcephaly and similar minor dysmorphisms (Fig. 1a, c, e, Supplementary Fig. 1b, Supplementary Case reports, Supplementary Movie 1, Supplementary Table 1, online resource, for detailed information on 18 cases). Ten of these individuals passed away early, with the majority before the age of 3.5 years. In six families, at least seven already deceased siblings had a similar phenotype but could not be investigated. Two families were of Indian descent (both with ancestors from regions currently belonging to Pakistan), living in Canada (family 2) and the USA (family 3), with the remaining families from Oman (family 4, originally from Pakistan), Pakistan (family 5, family 13), Iran (families 6, 7, 8 and 11), UAE (family 9), Saudi Arabia (family 10) and India (family 12). Two additional cases in family 14 from Oman and family 15 from India were identified presenting with intractable seizures and microcephaly, but no detailed medical information could be obtained at this point.

Having identified at least 22 individuals with an almost identical clinical phenotype and an identical homozygous variant in the same gene led us to pursue *UGP2* as a candidate gene for a new genetic form of DEE. *UGP2* is highly expressed in various brain regions (Fig. 1f), and also widely expressed amongst other tissues, including liver and muscle according to the data from the GTEx portal [23] (Supplementary Fig. 1d, online resource). The (chr2:64083454A>G) variant is predicted to cause a missense variant (c.34A>G, p.Met12Val) in *UGP2* isoform 1 (NM_006759), and to cause a translation start loss (c.1A>G, p.?.) of *UGP2* isoform 2 (NM_001001521), referred to as long and short isoforms, respectively. The variant has not been reported in the Epi25 web browser [31], ClinVar [54], LOVD [37], Exome Variant Server [33], DECIPHER [35], GENESIS [40], GME variome [90] or Iranome databases [34], is absent from our in-house data bases and is found only 15 times in a heterozygous, but not homozygous, state in the 280,902 alleles present in *gnomAD* (MAF: 0.00005340) [56]. In the *GeneDx* unaffected adult cohort, the variant was found heterozygous 10 times out of 173,502 alleles (MAF: 0.00005764), in the ~ 10,000 exomes of the Queen Square Genomic Center database two heterozygous individuals were identified, and out of 45,921 individuals in the *Centogene* cohort, 10 individuals are heterozygous for this variant. The identified variant has a CADD score (v1.4) of 19.22 [81] and Mutation Taster [89] predicted this variant as disease causing. The nucleotide is strongly conserved over multiple species (Fig. 1 g). Analysis of WES data from 5 patients did provide evidence of a shared ROH between patients from different families (including the Dutch family), indicating that this same variant might represent an ancient mutation that originated some 26 generations ago (Supplementary Fig. 1c, online resource). Interestingly, since most families originally came from regions of India, Pakistan and Iran, overlapping with an area called Balochistan, this could indicate that the mutation has originated there around 600 years ago. As Dutch traders settled in that area in the seventeenth century, it is tempting to speculate that this could explain the co-occurrence of the variant in these distant places [1].

### Short UGP2 isoform is predominantly expressed in brain and absent in patients with ATG mutations

**Fig. 2:**
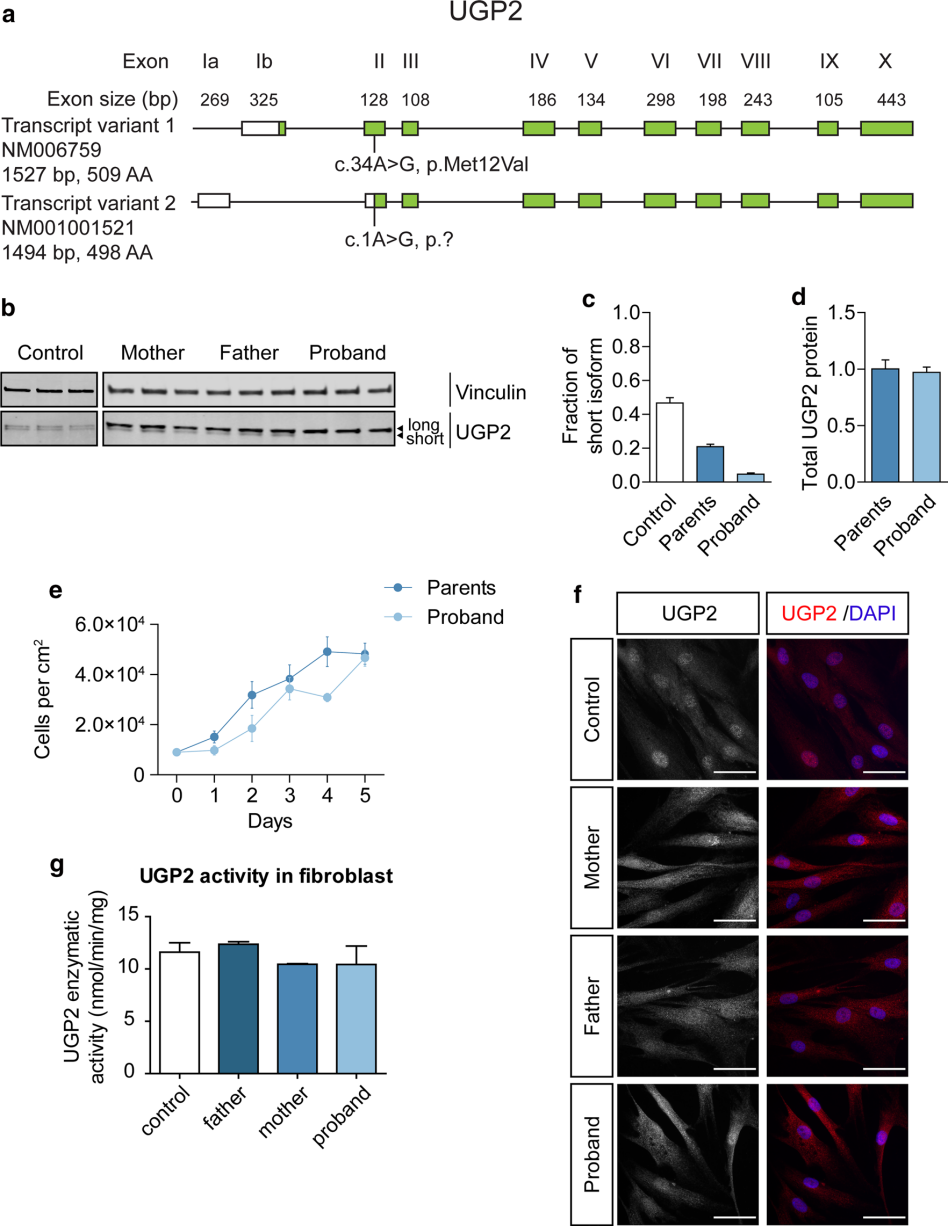
UGP2 homozygous variant leads to a loss of the shorter protein isoform in patient fibroblasts. **a** Schematic drawing of the human *UGP2* locus, with both long and short transcript isoforms. Boxes represent exons, with coding sequences indicated in green. The location of the recurrent mutation is indicated in both transcripts. **b** Western blotting of cellular extracts derived from control fibroblasts and fibroblasts obtained from family 1, detecting the housekeeping control vinculin and UGP2. Note the two separated isoforms of UGP2 that have a similar intensity in wild-type cells. The shorter isoform is less expressed in fibroblasts from heterozygous parents and absent in fibroblasts from the affected proband. **c** Western blot quantification of the fraction of short UGP2 protein isoform compared to total UGP2 expression in control, parental heterozygous and proband homozygous fibroblasts, as determined in three independent experiments. Error bars represent SEM. **d** Western blot quantification of total UGP2 protein levels, as determined by the relative expression to the housekeeping control vinculin. Bar plot showing the results from three independent experiments. Error bars represent SEM; no significant differences were found between parents and proband, *t* test, two tailed. **e** Cell proliferation experiment of fibroblasts from heterozygous parents and homozygous proband from family 1, during a 5-day period, determined in three independent experiments. Error bars represent SEM. **f** Immunocytochemistry on cultured control and UGP2 heterozygous and homozygous mutant fibroblasts derived from family 1, detecting UGP2 (red). Nuclei are stained with DAPI. Scale bar 50 µm. **g** Enzymatic activity of UGP2 in control and UGP2 heterozygous and homozygous mutant fibroblasts derived from family 1. Shown is the mean of two independent experiments. Error bars represent SEM; no significant differences were found, unpaired *t* test, two tailed

Both UGP2 isoforms only differ by 11 amino acids at the N-terminal (Fig. 2a) and are expected to be functionally equivalent [38]. To investigate how the A>G variant may cause DEE, we first obtained fibroblasts from individual 1 (homozygous for the A>G variant) and her heterozygous parents and analyzed the isoform expression by Western blotting (Fig. 2b). Whereas the two isoforms were equally expressed in wild-type fibroblasts, the expression of the shorter isoform was diminished to ~ 25% of total UGP2 in heterozygous parents, both of individual 1 (Fig. 2b, c) and of individuals 2 and 3 (Supplementary Fig. 2a, b, online resource), and was absent in cells from the affected individual 1 (Fig. 2b, c; fibroblasts of the affected children in family 2 or other families were not available). Total UGP2 levels were not significantly different between the affected child and her parents, or between parents and wild-type controls (Fig. 2d, Supplementary Fig. 2c, online resource). This indicates that the long isoform harboring the Met12Val missense variant is upregulated in fibroblast when the short isoform is missing. Moreover, this indicates that Met12Val does not affect the stability of the long isoform at the protein or transcript level (Supplementary Fig. 2d–f, online resource). RNA-seq on peripheral blood samples of family 1 did not identify altered splicing events of *UGP2* and the global transcriptome of the proband was not different from her parents, although only a limited analysis could be performed as only a single sample was available for each individual (Supplementary Fig. 2g, h, online resource). Both homozygous and heterozygous fibroblasts had a similar proliferation rate compared to wild-type fibroblasts (Fig. 2e, Supplementary Fig. 2i, online resource), and immunocytochemistry confirmed a similar subcellular localization of UGP2 in mutant and wild-type cells (Fig. 2f). We then measured the enzymatic activity of UGP2 in wild type, heterozygous and homozygous fibroblasts, and found that mutant fibroblast had a similar capacity to produce UDP-glucose in the presence of exogenously supplied glucose-1-phosphate and UTP (Fig. 2g). Altogether, this indicates that the long UGP2 isoform harboring the Met12Val missense change is functional and is, therefore, unlikely to contribute to the patient phenotype.

**Fig. 3:**
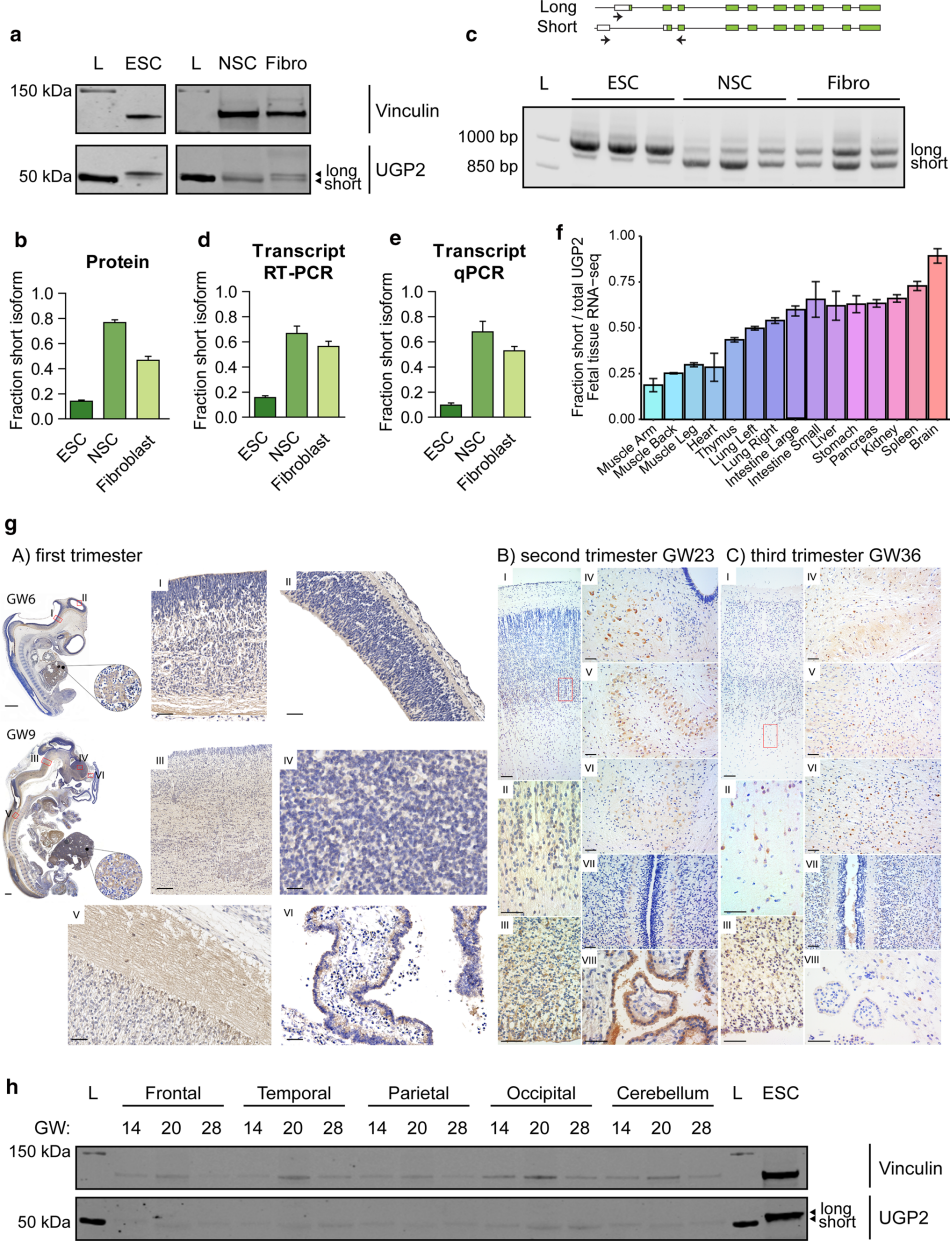
UGP2 short isoform is predominant in brain-related cell types. **a** Western blotting showing UGP2 expression in H9 human embryonic stem cells (ESCs), H9-derived neural stem cells (NSCs) and fibroblasts (Fibro). Vinculin is used as a housekeeping control. Note the changes in relative expression between the two UGP2 isoforms in the different cell types. L, ladder. **b** Western blot quantification of the fraction of short UGP2 protein isoform compared to total UGP2 expression, as determined in three independent experiments. Error bars represent SEM. **c** Multiplex RT-PCR of ESCs, NSCs and fibroblasts, showing a similar variability in isoform expression at the transcript and at the protein level. Each cell line was tested in triplicates. **d** Quantification of the fraction of the short *UGP2* transcript isoform compared to total *UGP2* expression, from the multiplex RT-PCR from **c**. Error bars represent SEM. **e** Quantification of the fraction of short *UGP2* transcript isoform compared to total *UGP2* expression by qRT-PCR in three independent experiments. Error bars represent SEM. **f** Ratio of RNA-seq reads covering the short transcript isoform compared to the total reads (covering both short and long isoforms), in multiple fetal tissues. In RNA-seq samples derived from brain, virtually all UGP2 expressions come from the short isoform. Error bars represent SD. **g** Immunohistochemistry detecting UGP2 in human fetal brains from the first, second and third trimester (gestational week (GW) 6, 9, 23 and 36). See text for details. **h** Western blotting detecting UGP2 in various human brain regions at weeks 14, 20 and 28 of gestation, showing the virtual absence of the long isoform expression in fetal brain. Vinculin is used as a housekeeping control. *L* ladder

As the A>G variant results in a functional long UGP2 isoform but abolishes the translation of the shorter UGP2 isoform, we next investigated whether the ratio between short and long isoform differs amongst tissues. If so, the homozygous A>G variant would lead to depletion of UGP2 in tissues where mainly the short isoform is expressed, possibly below a threshold that is required for normal development or function. Western blotting on cellular extracts derived from wild-type H9 human embryonic stem cells (ESCs), commercially acquired H9-derived neural stem cells (NSCs) and fibroblasts (Fig. 3a) showed that, whereas the ratio between short and long isoform in fibroblasts was around 0.5, in ESCs it was 0.14 and in NSCs 0.77, indicating that the shorter UGP2 isoform is the predominant one in NSCs (Fig. 3b). A similar trend was observed when assessing the transcript level, both by multiplex RT-PCR and qRT-PCR, using primers detecting specifically the short and long transcript isoform (Fig. 3c–e). This indicates that differential isoform expression between cell types is regulated at the transcriptional level, possibly hinting at tissue-specific regulatory elements driving isoform expression. We next analyzed RNA-seq data from human fetal tissues [46, 83, 94, 118] to determine the fraction of reads covering short versus total *UGP2* transcripts (Fig. 3f). This showed that in human fetal brain the short transcript isoform is predominantly expressed. To gain more insight into the cell type-specific expression of UGP2, we performed immunohistochemistry on human fetal brain tissues from the first to third trimester of pregnancy (Fig. 3g). In the first trimester, we found pale labeling of neuropil in the proliferative neuroepithelium of the hypothalamic, cortical, mesencephalic and thalamic regions (Fig. 3g-A/I, II, III, IV), as well as the marginal zone of the spinal cord (Fig. 3g-A/V) and cuboidal epithelial cells of choroid plexus (Fig. 3g-A/VI). During the second trimester, UGP2 positivity was detected in neurons from the subplate region of the cerebral cortex (Fig. 3g-B/I, II) and still in some of the cells in the neuroepithelium and subventricular zone (Fig. 3g-B/III). Almost the same pattern of UGP2 distribution was found in the cerebral cortex of fetuses from the third trimester. Also, we found clear cytoplasmatic UGP2 expression in neurons from mesencephalic, inferior olivary and cerebellar nuclei during the second (Fig. 3g-B/IV, V, and VI) and third trimester, respectively (Fig. 3g-C/IV, V). In the white matter of the cerebellum in the third trimester, we identified single-positive glial cells (Fig. 3g-C/VI). In the cerebellar cortex, we did not find specific positivity of cells on UGP2 (Fig. 3g-B, C/VII). Cuboidal epithelial cells of choroid plexus preserved UGP2 positivity during the second trimester (Fig. 3g-B/VIII) but lost it in the third trimester (Fig. 3g-C/VIII). Together this indicates that UGP2 can be detected in a broad variety of cell types during brain development. On Western blotting, we noticed preferential expression of the shorter UGP2 isoform in the developing cortex and cerebellum from gestational weeks 14, 20 and 28 (Fig. 3h) and in the frontal cortex of brains from weeks 21 and 23 (Supplementary Fig. 2j, online resource). Together, this supports the hypothesis that the DEE phenotype in patients is caused by a major loss of functional UGP2 in the brain, as the short isoform represents virtually all UGP2 produced in this tissue.

### Lack of the short UGP2 isoform leads to transcriptome changes upon differentiation into neural stem cells

To model the disease in vitro, we first engineered the homozygous A>G mutation in H9 ESCs to study the mutation in a patient independent genetic background and compare it to isogenic parental cells. We obtained two independent clones harboring the homozygous A>G change (referred to as knock-in, KI, mutant) and two cell lines harboring an insertion of an additional A after nucleotide position 42 of *UGP2* transcript 1 (chr2:64083462_64083463insA) (Supplementary Fig. 3a, b, online resource) (referred to as knockout, KO). This causes a premature stop codon at amino acid position 47 (D15Rfs*33), leading to nonsense-mediated mRNA decay and complete absence of UGP2 protein (Supplementary Fig. 3c, online resource). All derived ESCs had a normal morphology and remained pluripotent as assessed by marker expression (Supplementary Fig. 3d, e, online resource), indicating that the absence of UGP2 in ESCs is tolerated, in agreement with genome-wide LoF CRISPR screens which did not identify *UGP2* as an essential gene in ESCs [66, 119]. We differentiated wild type, KI and KO ESCs into NSCs, using dual SMAD inhibition (Supplementary Fig. 4a–c, online resource). Wild-type cells could readily differentiate into NSCs, having a normal morphology and marker expression, whereas differentiation of KI and KO cells was more variable and not all differentiations resulted in viable, proliferating NSCs. KO cells could not be propagated for more than five passages under NSC culture conditions (data not shown), which could indicate that the total absence of UGP2 protein is not tolerated in NSCs. When assessed by Western blotting, total UGP2 protein levels were reduced in KI cells and depleted in KO cells compared to wild type (Supplementary Fig. 4d, e, online resource).

**Fig. 4:**
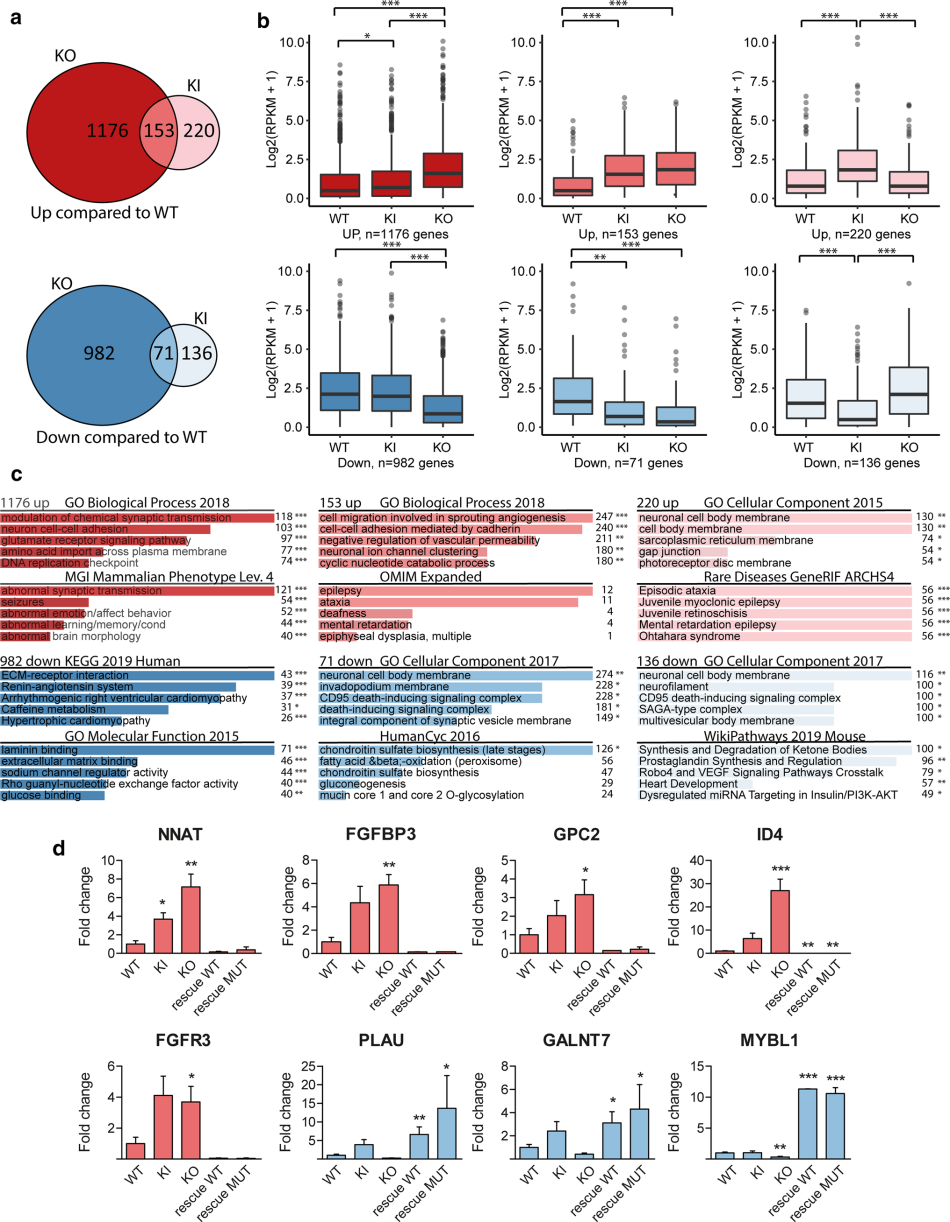
RNA-seq of UGP2 mutant H9-derived neural stem cells. **a** Venn diagram showing the overlap between differentially expressed genes in UGP2 KO or KI NSCs that are upregulated (upper panel, genes with FDR < 0.05 and LogFC > 1) or downregulated (lower panel, genes with FDR < 0.05 and LogFC < -1) compared to wild-type NSCs. **b** Box plot showing the distribution of gene expression levels [in Log2(RPKM + 1)] from RNA-seq for the groups of genes displayed in **a**, in wild type, UGP2 KI or KO NSCs. Boxes are IQR; line is median; and whiskers extend to 1.5 × the IQR (*p < 0.05; **p < 0.01, ***p < 0.001, unpaired *t* test, two tailed). **c** Enrichment analysis using Enrichr [52] of up- or downregulated genes in NSCs from **a** for selected gene ontology sets, showing the five most enriched terms per set. Combined score and *p* value calculated by Enrichr are depicted (*p < 0.05; **p < 0.01; ***p < 0.001). **d** qRT-PCR validation of differentially expressed genes from RNA-seq in wild type, UGP2 KI, UGP2 KO NSCs and KO NSCs rescued with either WT or MUT (Met12Val) transcript isoform 1, at p5 of NSC differentiation. Bar plot showing the mean fold change for the indicated genes compared to wild type, normalized for the housekeeping gene *TBP*. Results of two biological and two independent technical replicates are plotted. Colors match the Venn diagram group to which the tested genes belong, from **a**. Error bars represent SEM; (*p < 0.05; **p < 0.01, ***p < 0.001, unpaired *t* test, one-tailed)

Next, we performed RNA-seq of wild type, KI and KO ESCs and NSCs to assess how depletion of UGP2 upon NSC differentiation would impact the global transcriptome (Fig. 4, Supplementary Fig. 5, Supplementary Table 2, online resource). In agreement with normal proliferation and morphology of KI and KO ESCs, all ESCs shared a similar expression profile of pluripotency-associated genes and only few genes were differentially expressed between the three genotypes (Supplementary Fig. 5c, Supplementary Table 3, online resource). This indicates that the absence of UGP2 in ESCs does not lead to major transcriptome alterations despite the central role of this enzyme in metabolism. Upon differentiation, cells from all genotypes expressed NSC markers (Supplementary Fig. 5f, online resource), but when comparing wild type and KO cells, we observed noticeable changes that were less pronounced in KI NSCs but still followed a similar trend (Fig. 4a, b, Supplementary Fig. 5d, e, online resource). Gene enrichment analysis showed that genes downregulated in KO and KI cells were implicated in processes related to the extracellular matrix, cell–cell interactions and metabolism, while genes upregulated in KO and KI cells were enriched for synaptic processes and genes implicated in epilepsy (Fig. 4c, Supplementary Table 4, online resource). Both KO and KI cells showed an upregulation of neuronal expressed genes, indicating a tendency to differentiate prematurely. To validate RNA-seq findings, we tested several genes by qRT-PCR in wild type, KI and KO cells (Fig. 4d). We also included KO rescue cells, in which we had restored the expression of either the wild type or the mutant UGP2 long isoform, leading each to an approximately fourfold UGP2 overexpression at the NSC state compared to WT (Supplementary Fig. 4f, online resource). Amongst the tested genes was *NNAT*, which showed a significant upregulation in KI and KO cells, which was rescued by the restoration of UGP2 expression in KO NSCs. *NNAT* encodes neuronatin that stimulates glycogen synthesis by upregulating glycogen synthase and was previously found to be upregulated in Lafora disease. This lethal teenage-onset neurodegenerative disorder presenting with myoclonic epilepsy is caused by mutations in the ubiquitin ligase malin, leading to accumulation of altered polyglucosans [107]. Malin can ubiquitinate neuronatin leading to its degradation. As reduced UGP2 expression might impact glycogen production, it seems plausible that this results in compensatory *NNAT* upregulation and in downstream aberrations contributing to the patient phenotypes. Indeed, neuronatin upregulation was shown to cause increased intracellular Ca^2+^ signaling, ER stress, proteasomal dysfunction and cell death in Lafora disease [92, 93], and was shown to be a stress-responsive protein in the outer segment of retina photoreceptors [91, 95]. Another interesting gene upregulated in KI and KO NSCs and downregulated in rescue cell lines was the autism candidate gene *FGFBP3* [87]. This secreted proteoglycan that enhances FGF signaling is broadly expressed in brain [60], and functions as an extracellular chaperone for locally stored FGFs in the ECM, thereby influencing glucose metabolism by regulating rate-limiting enzymes in gluconeogenesis [102]. Other potentially relevant genes displaying the same expression trend were the heparan sulfate proteoglycan *GPC2* (a marker of immature neurons [64, 72]), the helix–loop–helix transcription factor *ID4* (a marker of postmitotic neurons [29]), and the signaling molecule *FGFR3* that has been implicated in epilepsy [73]. Genes downregulated in KO cells and upregulated in rescue cells included urokinase-type plasminogen activator *PLAU* (deficiency in mouse models increases seizure susceptibility [53]), the glycoprotein *GALNT7* (upregulation of which has been found to promote glioma cell invasion [45]) and the brain tumor gene *MYBL1* (that has been shown to be regulated by *O*-linked *N*-acetylglucosamine [42]. Similar expression changes were observed in NSCs differentiated from induced pluripotent stem cells (iPSCs) that we had generated from family 1 (Supplementary Fig. 6, online resource). Together, RNA-seq showed that whereas the absence of UGP2 is tolerated in ESCs, its complete absence or reduced expression results in global transcriptome changes in NSCs, with many affected genes implicated in DEE-relevant pathways.

### Absence of short UGP2 isoform leads to metabolic defects in neural stem cells

**Fig. 5:**
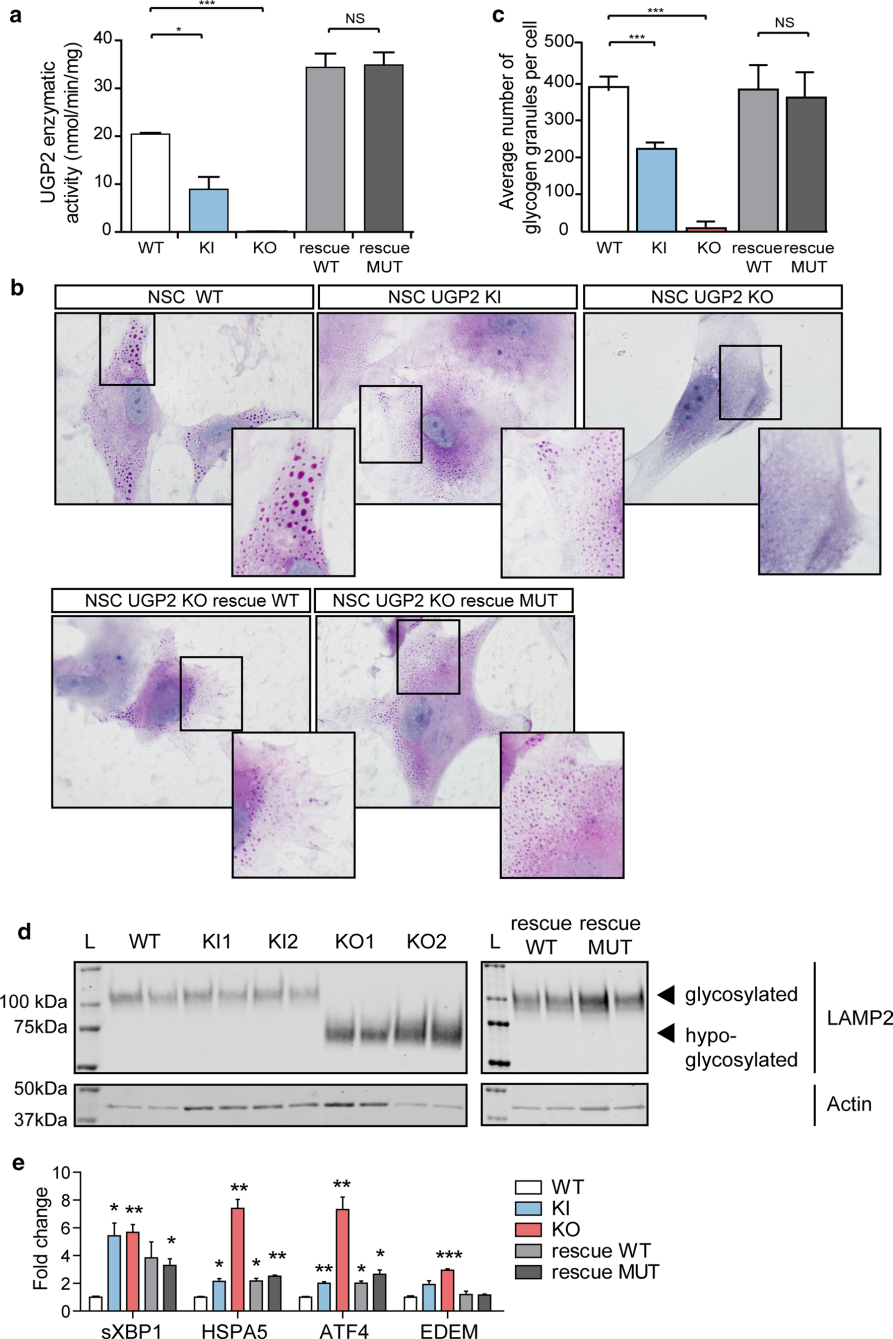
Metabolic changes upon UGP2 loss. **a** UGP2 enzymatic activity in WT, UGP2 KI, KO and KO NSCs rescued with WT or MUT (Met12Val) isoform 1 of UGP2. Bar plot showing the mean of two replicate experiments, error bar is SEM. *p < 0.05; ***p < 0.001, unpaired *t* test, two tailed. **b** Representative pictures of PAS staining in WT, KI, KO and rescued NSCs. Nuclei are counterstained with hematoxylin (blue). Inserts show zoom-in of part of the cytoplasm. Note the presence of glycogen granules in WT NSCs, their diminished number in KI NSCs, their absence in KO NSCs and their reappearance upon rescue with WT or MUT (Met12Val) isoform 1 of UGP2. **c** Quantification of the number of glycogen granules per cell in WT, UGP2 KI, KO and rescued NSCs, after 48 h culture under low-oxygen conditions. Shown is the average number of glycogen granules per cell, *n* = 80–100 cells per genotype. Error bars represent the SD. ***p < 0.001, unpaired *t* test, two tailed. **d** Western blotting detecting LAMP2 (upper panel) and the housekeeping control actin (lower panel) in cellular extracts from H9-derived NSCs that are WT, UGP2 KI, KO and KO cells rescued with WT or MUT (Met12Val) isoform 1 of UGP2. Glycosylated LAMP2 runs at ~ 110 kDa, whereas hypo-glycosylated LAMP2 is detected around 75 kDa. The absence of changes in LAMP2 glycosylation in KI cells is likely explained by a non-complete isoform switch upon in vitro NSC differentiation, resulting in residual UGP2 levels (see Supplementary Fig. 5d, online resource). **e** qRT-PCR expression analysis for UPR marker genes (spliced *XBP1*, *HSPA5*, *ATF4* and *EDEM*) in WT, KI, KO and rescued NSCs. Shown is the mean fold change for the indicated genes compared to wild type, normalized for the housekeeping gene *TBP*. Results of two biological and two independent technical replicates are plotted, from two experiments. Error bars represent SEM; *p < 0.05; **p < 0.01, ***p < 0.001, unpaired *t* test, two tailed

To investigate how reduced UGP2 expression levels in KO and KI cells would impact NSC metabolism, we investigated the capacity to produce UDP-glucose in the presence of exogenously supplied glucose-1-phosphate and UTP. KO NSCs showed a severely reduced ability to produce UDP-glucose (Fig. 5a). This reduction was rescued by ectopic overexpression of both long wild type and long mutant UGP2. KI cells showed a slightly reduced activity in ESCs (Supplementary Fig. 7a, online resource), but a more strongly reduced activity in NSCs compared to wild type (Fig. 5a), correlating with total UGP2 expression levels (Supplementary Fig. 4d, e, online resource). Surprisingly, contrary to KO NSCs, KO ESC showed some residual capacity to produce UDP-glucose despite the complete absence of UGP2 (Supplementary Fig. 7a, online resource). This could indicate that a yet to be identified enzyme can partially take over the function of UGP2 in ESCs but not NSCs, which might explain the lack of expression changes in this cell type upon UGP2 loss. iPSCs showed similar results (Supplementary Fig. 7b, online resource). We next assessed the capacity to synthesize glycogen under low-oxygen conditions by PAS staining, as it was previously shown that hypoxia triggers increased glycogen synthesis [78]. As expected, wild-type ESCs cultured for 48 h under hypoxia showed an intense cytoplasmic PAS staining in most cells (Supplementary Fig. 7c, d, online resource), while KO ESCs showed a severely reduced staining intensity. This indicates that under hypoxia conditions, the residual capacity of ESC to produce UDP-glucose in the absence of UGP2 is insufficient to produce glycogen. KI ESCs were indistinguishable from wild type (Supplementary Fig. 7d, online resource). At the NSC state, many KO cells kept at low-oxygen conditions for 48 h died (data not shown) and those KO cells that did survive were completely depleted from glycogen granules (Fig. 5b, c). This could be rescued by overexpression of both wild type and mutant long UGP2 isoform. KI NSCs showed a more severe reduction in PAS staining compared to the ESC state (Fig. 5b, c), and we observed similar findings in patient iPSC-derived NSCs (Supplementary Fig. 7e, online resource). Together, this further indicates that upon neural differentiation the isoform expression switch renders patient cells depleted of UGP2, leading to a reduced capacity to synthesize glycogen. This can directly be involved in the DEE phenotype, as, besides affecting energy metabolism, reduction of glycogen in brain has been shown to result in (1) impairment of synaptic plasticity [30]; (2) reduced clearance of extracellular potassium ions leading to neuronal hypersynchronization and seizures [22, 63, 117]; (3) altered glutamate metabolism [88]. To investigate how reduced UDP-glucose levels would impact on glycosylation, we next investigated glycosylation levels by means of LAMP2, a lysosomal protein known to be extensively glycosylated both by *N*-linked and *O*-linked glycosylation [114]. We found that KO NSCs show hypoglycosylation of LAMP2 that is rescued by the overexpression of both WT and mutant long isoform (Fig. 5d). In contrast, in ESCs, no glycosylation defects were noticed (Supplementary Fig. 7f, online resource). Finally, we investigated whether the absence of UGP2, affecting protein glycosylation, could induce ER stress and thus unfolded protein response (UPR). Whereas in ESCs, the absence of UGP2 did not result in a detectable effect on UPR markers (Supplementary Fig. 7g, online resource), in NSCs we noticed an increased expression of these genes both in KO and in KI cells (Fig. 5e). This indicates that NSCs having UGP2 levels under a certain threshold are more prone to ER stress and UPR. In agreement with this, we did not observe upregulation of UPR markers in patient-derived fibroblast, which have similar total UGP2 expression levels compared to controls (Supplementary Fig. 7h, online resource). Together this indicates that upon differentiation to NSCs, KI cells become sufficiently depleted of UGP2 to have reduced synthesis of UDP-glucose, leading to defects in glycogen synthesis and protein glycosylation and to the activation of UPR response. Alterations of these crucial processes are likely to be implicated in the pathogenesis leading to increased seizure susceptibility, altered brain microstructure and progressive microcephaly.

### ugp2a and ugp2b double mutant zebrafish recapitulate metabolic changes during brain development, have an abnormal behavioral phenotype, visual disturbance, and increased seizure susceptibility

**Fig. 6:**
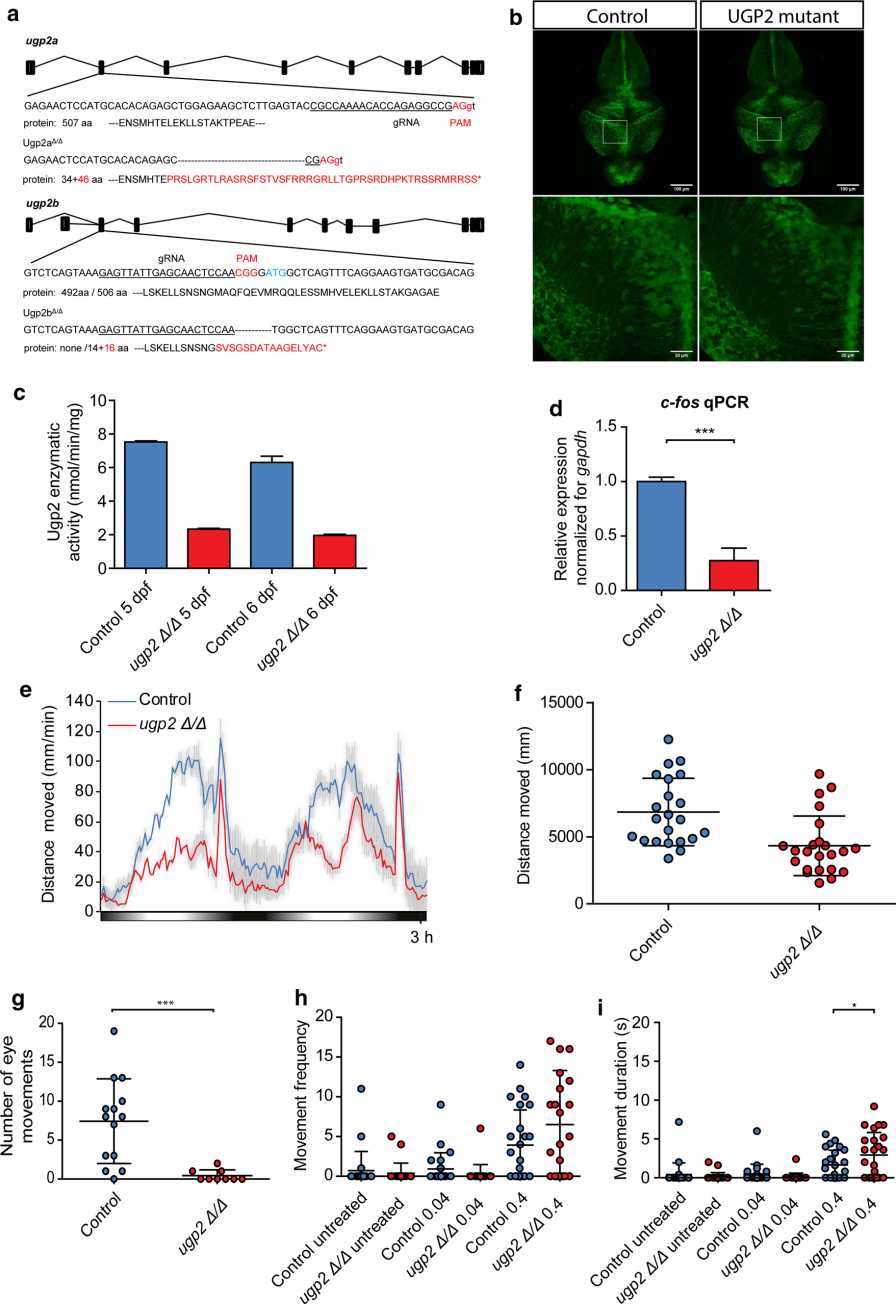
Zebrafish disease modeling. **a** Schematic drawing of the *ugp2a* and *ugp2b* loci in zebrafish and the generated mutations. **b** Confocal images (maximum projection of confocal Z-stacks) of the brain of wild type (left) and *ugp2a*^Δ/Δ^; *ugp2b*^Δ/Δ^ mutant zebrafish larvae (right), both in an *slc1a2b*-citrine reporter background, at 4 days post-fertilization (dpf). The lower panels are higher magnifications of the boxed regions indicated in the upper panels. Scale bar in upper panel is 100 µm, in lower panel 20 µm. In upper panel, *Z* = 45 with step size 4 µm; in lower panel, *Z* = 30 with step size 2 µm. **c** Enzymatic activity in *ugp2* double mutant zebrafish larvae at 4 and 5 dpf, compared to wild-type age-matched controls, showing reduced Ugp2 enzyme activity in double mutant zebrafish. **d** qRT-PCR for the neuronal activity marker *c*-*fos* in wild type and *ugp2* double mutant larvae at 3 dpf. For each group, 2 batches of 12 larvae were pooled. Shown is the mean fold change for the indicated genes compared to wild type, normalized for the housekeeping gene *gapdh*. Error bars represent SEM; ***p < 0.001, unpaired *t* test, two tailed. **e** Representative graph of a locomotion assay showing the total distance moved by larvae during the dusk–dawn routine (total time: 3 h 12 min), *n* = 24 larvae per genotype. Gray shading shows the standard error of the mean. **f** Quantification of the total distance moved throughout the experiment from **e** excluding the dark period. **g** Quantification of the number of observed spontaneous eye movements during a 2-min observation in wild type and *ugp2* double mutant larvae at 4 dpf. Each dot represents one larva; shown is the average and SD; ***p < 0.001, *t* test, two tailed. **h** Quantification of the frequency of movements at a speed of > 15 mm/s, for wild-type control and *ugp2* double mutant zebrafish larvae at 4 dpf, treated with mock control or with 0.04 nM or 0.4 nM 4-AP during a 35-min observation. Each dot represents a single larva; results of two experiments are shown, within total 24 larvae per condition. **i** Quantification of the movement duration at a speed of > 15 mm/s, for wild-type control and *ugp2* double mutant zebrafish larvae at 4 dpf, treated with mock control or with 0.04 nM or 0.4 nM 4-AP during a 35-min observation. Each dot represents a single larva; results of two experiments are shown, with in total 24 larvae per condition. *p < 0.05, two-way ANOVA with Bonferoni pos*t* test

Finally, to model the consequences of the lack of UGP2 in vivo, we generated zebrafish mutants for both *ugp2a* and *ugp2b*, the zebrafish homologs of *UGP2*, using CRISPR–Cas9 injections in fertilized oocytes in a background of a radial glia/neural stem cell reporter [51]. Double homozygous mutant lines having frameshift deletions for both genes confirmed by Sanger sequencing could be generated but the only viable combination, obtained with *ugp2a* loss, created a novel ATG in exon 2 of ugp2b, leading to a hypomorphic allele (Fig. 6a). Homozygous *ugp2a/b* mutant zebrafish had a normal gross morphology of brain and radial glial cells (Fig. 6b), showed a largely diminished activity to produce UDP-glucose in the presence of exogenously supplied glucose-1-phosphate and UTP (Fig. 6c), and showed a reduction in *c*-*fos* expression levels, indicating reduced global neuronal activity (Fig. 6d). To monitor possible spontaneous seizures, we performed video tracking experiments of developing larvae under light–dark cycling conditions at 5 days post-fertilization (dpf). Control larvae show increased locomotor activity under light conditions, and although *ugp2* double mutant larvae still responded to increasing light conditions, they showed a strongly reduced activity (Fig. 6e, f). This could indicate that their capability to sense visual cues is diminished, or that their tectal processing of visual input is delayed, resulting in reduced movements. Strikingly, upon careful inspection, we noticed that *ugp2* double mutant larvae did not show spontaneous eye movements, in contrast to age-matched control larvae (Fig. 6g, Supplemental Movies 2 and 3, online resource). Whereas we did not observe an obvious spontaneous epilepsy phenotype in these double mutant larvae, upon stimulation with 4-aminopyridine (4-AP), a potent convulsant, double mutant larvae showed an increased frequency and duration of movements at high velocity compared to controls, which might indicate an increased seizure susceptibility (Fig. 6h, i). Taken together, severely reduced Ugp2a/Ugp2b levels result in a behavior defect with reduced eye movements, indicating that also in zebrafish Ugp2 plays an important role in brain function.

### *UGP2 is an essential gene in humans and ATG mutations of tissue*-*specific isoforms of essential genes potentially cause more rare genetic diseases*

**Fig. 7:**
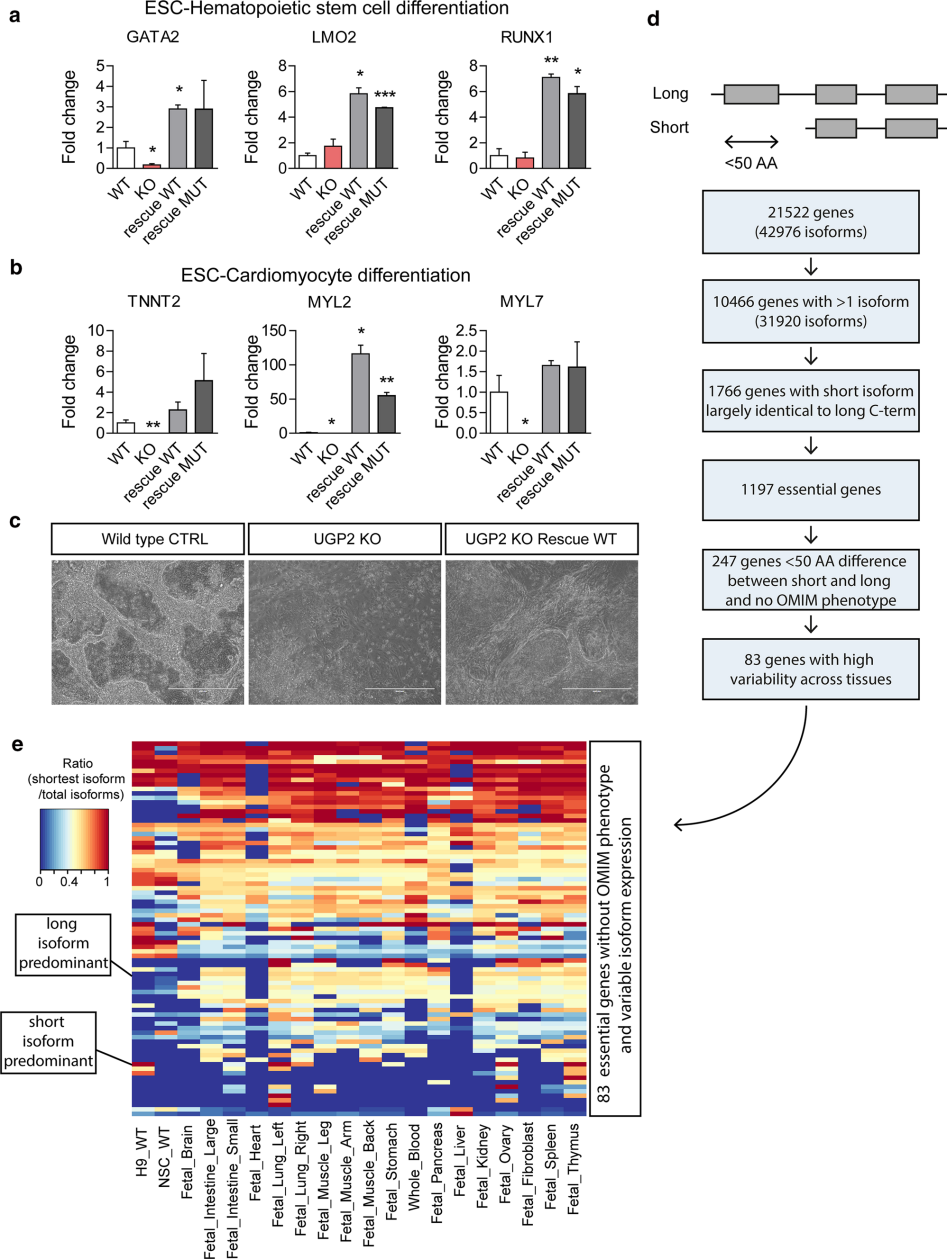
Essentiality of UGP2 and other disease candidate genes with a similar mutation mechanism. **a** qRT-PCR analysis of the hematopoietic stem cell markers *GATA2*, *LMO2* and *RUNX1*, after 12 days of differentiation of wild type, UGP2 KO and UGP2 KO rescue ESCs. Shown is the mean fold change for the indicated genes compared to wild type, normalized for the housekeeping gene *TBP*. Results of two biological and two technical replicates are plotted. Error bars represent SEM; *p < 0.05; **p < 0.01, ***p < 0.001, unpaired *t* test, two tailed. **b** As **a**, but now for cardiomyocyte differentiation at day 15, assessing expression of the cardiomyocyte markers *TNNT2*, *MYL2* and *MYL7*. **c** Bright-field image of cardiomyocyte cultures of wild type, UGP2 KO and rescue cells. Note the elongated organized monolayer structure cardiomyocytes capable of beating in wild type and rescue cells that are absent in KO cultures. Scale bar is 400 µm. **d** Scheme showing the homology search to identify genes with a similar structure as UGP2, where ATG-altering mutations could affect a tissue-specific isoform causing genetic disease. **e** Heat map showing the ratio of short isoform expression over total isoform expression from published RNA-seq data amongst 20 tissues for 83 out 247 essential genes that are not yet implicated in disease and in which the short and longer protein isoforms differ by less than 50 amino acids at the N-terminal

Several lines of evidence argue that UGP2 is essential in humans. First, no homozygous LoF variants or homozygous exon-covering deletions for *UGP2* are present in *gnomAD or GeneDx controls,* and homozygous variants in this gene are limited to non-coding changes, synonymous variants and five missense variants, together occurring only seven times homozygous (Supplementary Table 5, online resource). Also, no homozygous or compound heterozygous *UGP2* LoF variants were found in published studies on dispensable genes in human knockouts [70, 86, 99], or in the *Centogene* (*CentoMD®*) or *GeneDx* patient cohorts, encompassing together many thousands of individuals, further indicating that this gene is intolerant to loss-of-function in a bi-allelic state. In addition, no homozygous deletions of the region encompassing *UGP2* are present in DECIPHER [35] or ClinVar [54]. Second, *UGP2* has been identified as an essential gene using gene-trap integrations [17] and in CRISPR–Cas9 LoF screens in several human cell types [5, 15, 43, 113, 115]. Finally, studies in yeast [25, 26], fungus [58] and plants [21, 75, 116] consider the orthologs of *UGP2* as essential, and the absence of *Ugp2* in mice is predicted to be lethal [104]. In flies, homozygous UGP knockouts are lethal while only hypomorphic compound heterozygous alleles are viable but have a severe movement defect with altered neuromuscular synaptogenesis due to glycosylation defects [48]. To further investigate the essentiality of UGP2, we performed differentiation experiments of our WT, KO and rescue ESCs. Differentiation of KO ESCs into hematopoietic stem cells (HSCs) resulted in severe downregulation of *GATA2* compared to wild-type cells, and this was restored in rescue cell lines (Fig. 7a). GATA2 is a key transcription factor in the developing blood system, and knockout of *Gata2* is embryonic lethal in mice due to defects in HSC generation and maintenance [28, 106]. Differentiation of ESCs into cardiomyocytes similarly affected key marker gene expression in KO cells, and these changes were restored upon UGP2 rescue (Fig. 7b, c). Whereas WT ESCs could generate beating cardiomyocytes after 10 days, these were not seen in KO ESCs. Taken together this argues that the complete absence of UGP2 in humans is probably incompatible with life, a hypothesis that cannot be tested directly. However, if true, this could well explain the occurrence of the unique recurrent mutation in all cases presented herein. Given the structure of the *UGP2* locus (Fig. 2a), every LoF variant would affect either the long isoform, when located in the first 33 nucleotides of the cDNA sequence, or both the short and long isoforms when downstream to the ATG of the short isoform. Therefore, the short isoform start codon is the only mutational target that can disrupt specifically the short isoform. In this case, the Met12Val change introduced into the long isoform does not seem to disrupt UGP2 function to such an extent that this is intolerable and, therefore, allows development to proceed for most tissues. However, the lack of the short UGP2 isoform caused by the start codon mutation results in a depletion of functional UGP2 in tissues where normally the short isoform is predominantly expressed. In brain, this reduction diminishes total UGP2 levels below a threshold for normal development, causing a severe epileptic encephalopathy syndrome. Given the complexity of the human genome with 42,976 transcripts with RefSeq peptide IDs, perhaps also other genetic disorders might be caused by such tissue-restricted depletion of essential proteins. Using a computational homology search of human proteins encoded by different isoforms, we have identified 1766 genes that share a similar structure to the *UGP2* locus (e.g., a shorter protein isoform that is largely identical to the longer protein isoform, translated from an ATG that is contained within the coding sequence of the long isoform) (Fig. 7d). When filtering these genes for (1) those previously shown to be essential [10], (2) not associated with disease (e.g., no OMIM phenotype) and (3) those proteins where the shorter isoform is no more than 50 amino acids truncated at the N-terminal compared to the longer isoform, we identified 247 genes (Supplementary Table 6, online resource). When comparing the ratios of isoform-specific reads obtained from different fetal RNA-seq data [46, 83, 94, 118], we noticed that many of these genes show differential isoform expression amongst multiple tissues, with many genes showing either expression of the long or the short isoform in a particular tissue (Fig. 7e). Homozygous LoF variants or start codon altering mutations in these genes are rare in *gnomAD* (Supplementary Table 7, online resource), and it is tempting to speculate that mutations in start codons of these genes could be associated with human genetic diseases, as is the case for *UGP2*. Using mining of data from undiagnosed patients from our own exome database, the Queen Square Genomic Center database and those from *Centogene* and *GeneDx*, we found evidence for several genes out of the 247 having rare, bi-allelic variants affecting the start codon of one of the isoforms that could be implicated in novel disorders (*unpublished observations*) and give one such example in the Supplementary Note, online resource. Together, these findings highlight the relevance of mutations resulting in tissue-specific protein loss of essential genes for genetic disorders.

## Discussion

Here we describe a recurrent variant in 22 individuals from 15 families, affecting the start codon of the shorter isoform of the essential gene *UGP2* as a novel cause of a severe DEE. Using in vitro and in vivo disease modeling, we provide evidence that the reduction of *UGP2* expression in brain cells leads to global transcriptome changes, a reduced ability to produce glycogen, alterations in glycosylation and increased sensitivity to ER stress, which together can explain the phenotype observed in the patients. Most likely our findings in vitro underestimate the downstream effects in patient cells, as in fetal brain, the longer isoform expression is almost completely silenced and virtually all UGP2 come from the shorter isoform, which in patient cells cannot be translated. During our in vitro NSC differentiation, this isoform switch is less complete, leaving cells with the patient mutation with some residual UGP2. Strikingly, the clinical phenotype seems to be very similar in all cases, including intractable seizures, absence of developmental milestones, progressive microcephaly and a disturbance of vision, with retinal pigment changes observed in all patients who had undergone ophthalmological examination. Also, all patients seem to share similar, although mild, dysmorphisms, possibly making this condition a recognizable syndrome.

The involvement of *UGP2* in genetic disease is surprising. Given its central role in nucleotide-sugar metabolism it is expected that loss of this essential protein would be incompatible with life and, therefore, loss-of-function should not be found in association with postnatal disease. Our data argue that indeed a total absence of UGP2 in all cells is lethal, but that tissue-specific loss, as caused here by the start codon alteration of an isoform important for brain, can be compatible with postnatal development but still results in a severe phenotype. Given that any other LoF variant across this gene would most likely affect both protein isoforms, this could also explain why only a single mutation is found in all individuals. The fact that the Met12Val long isoform was able to rescue the full KO phenotype indicates that the missense change introduced to the long protein isoform does not affect UGP2 function. As other variants at this start codon, even heterozygous, are not found, possibly missense variants encoding for leucine, lysine, threonine, arginine or isoleucine (e.g., amino acids that would be encoded by alternative changes affecting the ATG codon) at this amino acid location in the long isoform could not produce a functional protein and are, therefore, not tolerated. Although start codon mutations have previously been implicated in disease [16, 19], there are no reports, to our knowledge, on disorders describing start codon alterations of other essential genes, leading to alterations of tissue-specific isoforms. Using a genome-wide homology search, we have identified a large list of other essential genes with a similar locus structure and variable isoform expression amongst tissues, where similar ATG-altering variants could affect tissue-relevant expression. An intriguing question is why evolution has resulted in a large number of genes encoding almost identical protein isoforms. It will be interesting to further explore the mutational landscape of these genes in cohorts of currently unexplained patients.

## Electronic supplementary material

Below is the link to the electronic supplementary material.

Supplemental Movie 1: affected individual from family 11 (MP4 21603 kb)

Supplemental Movie 2: wild type zebrafish eye movements (AVI 965 kb)

Supplemental Movie 3: Ugp2a/b double mutant zebrafish eye movements (AVI 1081 kb)

Supplementary Table 1: Extended clinical characteristics of 18 patients with homozygous UGP2 variants (XLSX 32 kb)

Supplementary Table 2: RNA-seq data used in this study (XLSX 15 kb)

Supplementary Table 3: differentially expressed genes (XLS 14057 kb)

Supplementary Table 4: enrichment analysis (XLSX 7124 kb)

Supplementary Table 5: * UGP2* variants in * gnomAD* (XLSX 224 kb)

Supplementary Table 6: genome-wide homology search results (XLS 41 kb)

Supplementary Table 7: *gnomAD* data of 247 disease candidate genes (XLS 1393 kb)

Supplementary file11 (PDF 475 kb)

Supplementary file12 (PDF 55596 kb)
