# Supplementary material for: Loss of UGP2 in brain leads to a severe epileptic encephalopathy, emphasizing that bi-allelic isoform-specific start-loss mutations of essential genes can cause genetic diseases
Source: Acta Neuropathol. 2019 Dec 9;139(3):415–42. doi: 10.1007/s00401-019-02109-6 (PMC7035241; doi:10.1007/s00401-019-02109-6)
Supplement: Supplementary file 11 — Supplementary file11 (PDF 475 kb) [file 401_2019_2109_MOESM11_ESM.pdf]

Supplementary Table 8: oligonucleotides used in this study

**Primer name**	**Sequence**
B484_Hs_XBP1s L	CTGAGTCCGAATCAGGTGCAG
B485_Hs_XBP1s R	ATCCATGGGGAGATGTTCTGG
B486_Hs_EDEM L	CAAGTGTGGGTACGCCACG
B487_Hs_EDEM R	AAAGAAGCTCTCCATCCGGTC
B488_Hs_HSPA5 L	TGTTCAACCAATTATCAGCAAACTC
B489_Hs_HSPA5 R	TTCTGCTGTATCCTCTTCACCAGT
B490_hATF4_fw	GTCCCTCCAACAACAGCAAG
B491_hATF4_rv	GCATGGTTTCCAGGTCATCT
239 hOCT3/4-S1165	GACAGGGGGAGGGGAGGAGCTAGG
240 hOCT3/4-AS1283	CTTCCCTCCAACCAGTTGCCCCAAAC
243 h NANOG-S	CAG CCC CGA TTC TTC CAC CAG TCC C
244 h NANOG-AS	CGG AAG ATT CCC AGT CGG GTT CAC C
245 hREX1-RT-S	CAG ATC CTA AAC AGC TCG CAG AAT
246 hREX1-RT-AS	GCG TAC GCA AAT TAA AGT CCA GA
794 hPAX6 fw	TCCACCCGGCAGAAGATTGTA
795 hPAX6 rv	TGTCTCGGATTTCCCAAGCAA
802 hGFAP fw	AGAGATCCGCACGCAGTATG
803 hGFAP rv	TCTGCAAACTTGGAGCGGTA
B455_UPG2_gRNA1_fw	CACCGAGCAAAGCAATGTCTCAAGA
B456_UPG2_gRNA1_rv	AAACTCTTGAGACATTGCTTTGCTC
B459_UGP2_c34G_ssODN	G*T*TTTAAGATCTTAGCAAAGCAGTGTC TCAAGATGGTGCTTCTCAGTTCCAAGAAG TC*A*T
B496_UGP2_hum_genotyp_fw	GCTGAAATTGGGAACCAGAA
B497_UGP2_hum_genotyp_rv	GGGGGTGGAACACTGTAGAA
c-Myc_Forward	TAA CTG ACT AGC AGG CTT GTC G
c-Myc_Reverse	TCC ACA TAC AGT CCT GGA TGA TGA TG
Klf4-Forward	TTC CTG CAT GCC AGA GGA GCC C
Klf4-Reverse	AAT GTA TCG AAG GTG CTC AA
hSOX2-S1430	GGG AAA TGG GAG GGG TGC AAA AGA GG
hSOX2-AS1555	TTG CGT GAG TGT GGA TGG GAT TGG TG
hGDF3-S243	CTT ATG CTA CGT AAA GGA GCT GGG
hGDF3-AS850	GTG CCA ACC CAG GTC CCG GAA GTT
hFGF4-RT-S	CTA CAA CGC CTA CGA GTC CTA CA
hFGF4-RT-AS	GTT GCA CCA GAA AAG TCA GAG TTG
hESG1-S40	ATA TCC CGC CGT GGG TGA AAG TTC
hESG1-AS259	ACT CAG CCA TGG ACT GGA GCA TCC
hTERT-S3234	CCT GCT CAA GCT GAC TCG ACA CCG TG
hTERT-AS3713	GGA AAA GCT GGC CCT GGG GTG GAG C
B163_tbp_fw	GGGGAGCTGTGATGTGAAGT
B164_tbp_rv	CCAGGAAATAATTCTGGCTCA

B453_UGP2_NM_001001521_5'UTR_fw	TTTCCCAGCTGCTAAAGGAA
B454_UGP2_NM_001001521_5'UTR_rv	GCCGAATGACTTCTTGGAAC
B447_UGP2_exon 3-4_fw	TGGATTTCGGAAGCTATTTCA
B448_UGP2_exon 3-4_rv	CCCCTGGCCTTTATCTTTTC
UGP2-201_F	TGTAAAACGACGGCCAGTGGAAGAGAGA CCTGCCCTGT
UGP2-202_F	TGTAAAACGACGGCCAGTAAAGTAGGGG CTGTGGGTGT
UGP2_R	CAGGAAACAGCTATGACCTGCTGAACAGT CAGATCCAGA
B707_NNAT_iso1_ex1-ex2_fw	TCATCATCGGCTGGTACATC
B708_NNAT_iso1_ex1-ex2_rv	CTGTGTCCCTGGAGGATTTC
B703_FGFBP3_ex1-ex2_fw	GTGCAGTCGTTCCCTCTACC
B704_FGFBP3_ex1-ex2_rv	TTCTCAGACCACGTTTGTCG
B717_ID4_ex2-ex3_fw	GAACAAGCAGGGCGACAG
B718_ID4_ex2-ex3_rv	CCCCTCCCTCTCTAGTGCTC
B689_PLAU_Ex10-Ex11_fw	CTGACCCACAGTGGAAAACA
B690_PLAU_Ex10-Ex11_rv	CCAGCTCACAATTCCAGTCA
B701_MYBL1_ex7-ex8_fw	TGTTCAGCCTACTTCTGCCTTT
B702_MYBL1_ex7-ex8_rv	TCATTCTCAGCTGACATAAGAAGC
B691_GALNT7_ex4-ex5_fw	GCACGAAGTATTGGTGCTCA
B692_GALNT7_ex4-ex5_rv	GGGAGCTACAAGTGGTGCAT
B727_FGFR3_ex2-ex3_fw	GCCTCCTCGGAGTCCTTG
B730_FGFR3_ex4-ex5_rv	CAGCAGCTTCTTGTCCATCC
B719_GPC2_ex1-ex2_fw	GCCCGGGGATATAGCTTAAA
B720_GPC2_ex1-ex2_rv	TCAGCCTCTGCTCTGTCTCA
gRNA sequence A2	GAGTACCGCCAAAACACCAG
gRNA sequence B1	ATTGAGCAACTCCAACGGGA
B743_c-Fos_zebra_fw	AACTGTCACGGCGATCTCTT
B744_c-Fos_zebra_rv	GCAGGCATGTATGGTTCAGA
B745_gapdh_zebra_fw	GTGGAGTCTACTGGTGTCTT
B746_gapdh_zebra_rv	GTGCAGGAGGCATTGCTTAC
B631_Ugp2a-in1-Fb	ATCCTGCTTTCCTGTGTGTG
B632_Ugp2a-in2-Rb	AGGTTAACTAGGCAGGTTAGGG
B466_UGP2b_fish_gen_fw	CAAGCCCAGTGATTGATGTG
B467_UGP2b_fish_gen_rv	GCAAAGCAAACAAGGCTCAT
B761_GATA2_ex2e3_fw	GCAACCCCTACTATGCCAAC
B762_GATA2_ex2e3_rv	GGGCTGTGCAACAAGTGTG
B757_LMO2_ex2ex3_fw	CGGCGCCTCTACTACAAACT
B758_LMO2_ex2ex3_rv	GAATCCGCTTGTCACAGGAT
B755_RUNX1_fw	ATCGCTTTCAAGGTGGTGG
B756_RUNX1_fw	CACTTCGACCGACAAACCT
B763_TNNT2_fw	ATGATGCATTTTGGGGGTTA
B764_TNNT2_rv	CAGCACCTTCCTCCTCTCAG
B765_MYL2_ex2ex3_fw	CAACGTGTTCTCCATGTTCG
B766_MYL2_ex2ex3_rv	GTCAATGAAGCCATCCCTGT

B767_MYL7_ex2ex3_fw	AACGTGGTTCTTCCAACGTC
B768_MYL7_ex2ex3_rv	AGGTCTGCCTTGCAGATGAT
B494_UGP2_Gib_short_UTR2_fw	GTCTCATCATTTTGGCAAAGTTTCCCAGCTGCTAAAGGAA
B495_UGP2_Gib_short_G_fw	GTCTCATCATTTTGGCAAAGGTGTCTCAAGATGGTGCTTCT
B460_UGP2_Gib_long_fw	GTCTCATCATTTTGGCAAAGATGTCGAGATTTGTACAAGATC
B461_UGP2_Gib_rev_STANDARD	GAGGGAGAGGGGCGGAATTTTCAGTGGTCCAAGATGCGAA
B462_UGP2_Gib_short_fw	GTCTCATCATTTTGGCAAAGATGTCTCAAGATGGTGCTTCT
B448_UGP2_exon 3-4_rv	CCCCTGGCCTTTATCTTTTC
B451_UGP2_exon 9-10_fw	TGCTTGAATTGGATCACCTC
