# Supplementary material for: Loss of UGP2 in brain leads to a severe epileptic encephalopathy, emphasizing that bi-allelic isoform-specific start-loss mutations of essential genes can cause genetic diseases
Source: Acta Neuropathol. 2019 Dec 9;139(3):415–42. doi: 10.1007/s00401-019-02109-6 (PMC7035241; doi:10.1007/s00401-019-02109-6)
Supplement: Supplementary file 12 — Supplementary file12 (PDF 55596 kb) [file 401_2019_2109_MOESM12_ESM.pdf]

Supplementary Materials, online resource

Loss of UGP2 in brain leads to a severe epileptic encephalopathy, emphasizing that bi-allelic isoform specific start-loss mutations of essential genes can cause genetic diseases

Elena Perenthaler, Anita Nikoncuk, Soheil Yousefi, Woutje M. Berdowski, Maysoon Alsagob, Ivan Capo, Herma C. van der Linde, Paul van den Berg, Edwin H. Jacobs, Darija Putar, Mehrnaz Ghazvini, Eleonora Aronica, Wilfred F.J. van IJcken, Walter G. de Valk, Evita Medici–van den Herik, Marjon van Slegtenhorst, Lauren Brick, Mariya Kozenko, Jennefer N. Kohler, Jonathan A. Bernstein, Kristin G. Monaghan, Amber Begtrup, Rebecca Torene, Amna Al Futaisi, Fathiya Al Murshedi, Renjith Mani, Faisal Al Azri, Erik-Jan Kamsteeg, Majid Mojarrad, Atieh Eslahi, Zaynab Khazaei, Fateme Massinaei Darmiyan, Mohammad Doosti, Ehsan Ghayoor Karimiani, Jana Vandrovcova, Faisal Zafar, Nuzhat Rana, Krishna K. Kandaswamy, Jozef Hertecant, Peter Bauer, Mohammed A. AlMuhaizea, Mustafa A. Salih, Mazhor Aldosary, Rawan Almass, Laila Al-Quait, Wafa Qubbaj, Serdar Coskun, Khaled O. Alahmadi, Muddathir H.A. Hamad, Salem Alwadaee, Khalid Awartani, Anas M. Dababo, Futwan Almohanna, Dilek Colak, Mohammadreza Dehghani, Mohammad Yahya Vahidi Mehrjardi, Murat Gunel, A. Gulhan Ercan-Sencicek, Gouri Rao Passi, Huma Arshad Cheema, Stephanie Efthymiou, Henry Houlden, Aida M. Bertoli-Avella, Alice S. Brooks , Kyle Retterer, Reza Maroofian, Namik Kaya, Tjakko J. van Ham and Tahsin Stefan Barakat

<u>Content</u>:

Supplementary Figure Legends

Supplementary Figures

Supplementary Case Reports

Supplementary Note

Supplementary References

Supplementary Figure Legends

Supplementary Figure 1, related to Figure 1:

a) Growth chart from individual 1 for length (left) and head circumference (right) in cm. Reference chart from the Dutch population are used (TNO) and regions between -2 and + 2 SD are shaded.
b) MRI studies of individual 6, individual 5 (at the age of 12 months), and individual 19 (at the age of 4 months), showing global brain atrophy.
c) ROH comparison between affected individuals from family 1, 4, 5, 6 and 7, carrying the homozygous chr2:64083454A>G mutation. The red line indicates the UGP2 variant, and the blue lines demark the shared ROH region between the individuals (chr2:60679942-65667235).
d) Violin plots showing distribution of gene expression (in TPM) amongst samples from the GTEx portal [1] for tissues and cell lines. Samples are sorted with the highest median TPM on the right. Outliers are indicated by dots.

Supplementary Figure 2, related to Figure 2:

a) Western blotting of cellular extracts derived from control fibroblasts or fibroblasts obtained from heterozygous parents of family 2, detecting the house keeping control vinculin or UGP2. Note the two separated isoforms of UGP2 that have a similar intensity in wild type cells. The shorter isoform shows reduced expression in fibroblasts from heterozygous parents.
b) Quantification of the fraction of the short UGP2 protein isoform compared to total UGP2 expression in control, and heterozygous fibroblasts from family 2, as determined in three independent experiments. Error bars represent SEM.
c) Western blot quantification of total UGP2 protein levels, as determined by the relative expression to the housekeeping control vinculin. Bar graph showing the results from three independent experiments. Error bars represent SEM; no significant differences between control and parent samples, unpaired t-test, two-tailed.
d) qRT-PCR analysis of total *UGP2* or the short isoform in fibroblast from heterozygous parents or homozygous proband from family 1, normalized for the housekeeping control *TBP*. The mean fold change compared to heterozygous parents of two biological replicates and two technical replicates is shown; error bars represent SEM no significant differences between control and parent samples, unpaired t-test, two-tailed.
e) Multiplex RT-PCR detecting relative expression of *UGP2* isoform 1 and isoform 2 in peripheral blood from family 1 and unrelated wild type controls.
f) Sanger sequencing of RT-PCR products from **e**), showing the expression of the homozygous and heterozygous chr2:64083454A>G *UGP2* variant in the index proband, her parents and an unrelated control.
g) Heat map showing genome-wide gene expression levels (in log2(RPKM+1)) in peripheral blood from heterozygous parents and homozygous proband from family 1.
h) Gene expression levels (in log2(RPKM+1)) from RNA-seq in peripheral blood for a selected number of genes involved in metabolism.

i) Cell proliferation experiment of fibroblast from heterozygous parents from family 2 and wild type controls, during a 5 days period. Error bars represent SEM, **= $p<0.01$, unpaired t-test, two-tailed.
j) Western blotting detecting UGP2 in human frontal cortex from week 21 and 23 of gestation, showing the virtual absence of the long isoform expression in fetal brain. Vinculin is used as a housekeeping control.

Supplementary Figure 3, related to Figure 4: generation of mutant *UGP2* H9 cell lines

a) Nucleotide sequence encompassing the ATG of *UGP2* transcript isoform 2. Indicated are the coding sequence, the location of the gRNA, PAM sequence and ssODN used to introduce the C.1A>G, p.? mutation.
b) Sanger sequencing traces of part of the UGP2 gene from wild type, *UGP2* knock-out (KO) and *UGP2* knock-in H9 ESCs (KI). The A at the start of the coding sequence of UGP2 isoform 2 (short isoform) is highlighted. The homozygous insertion of an additional A in knockout and the mutation into a G in knock-in cells are indicated.
c) Western blot detecting UGP2 and vinculin in wild type ESC, heterozygous and homozygous knockout and knock-in ESCs, as indicated. Note the complete loss of UGP2 in KO cells, and the loss of the short isoform in KI cells.
d) RT-qPCR detecting the pluripotency factors OCT4, NANOG and REX1 in H9 wild type, UGP2 knock-in (KI) and *UGP2* knock-out (KO) ESCs, normalized for the house keeping control *TBP*. Mean fold change compared to wild type of two biological replicates and three technical replicates is shown; error bars represent SEM, *= $p<0.05$, unpaired t-test, two-tailed.
e) Bright field image of a representative ESC colony from wild type parental and *UGP2* KO ESCs.

Supplementary Figure 4, related to Figure 4, NSC differentiation

a) Schematic drawing of the differentiation procedure, see online methods for details.
b) Bright field image showing representative pictures from ESCs and differentiated NSCs.
c) qRT-PCR analysis for pluripotency markers (*NANOG*, *OCT4* (*POU5F1*), *REX1*) and genes expressed in NSCs (*PAX6*, *GFAP*) in WT, UGP2 KO and KI differentiated NSCs at p1 and p5. Mean fold change compared to wild type of two biological replicates and two technical replicates is shown; error bars represent SEM.
d) Western blotting showing UGP2 expression in WT, UGP2 KI and KO differentiated NSCs. Vinculin is used as a housekeeping control.
e) Quantification of total UGP2 protein levels by Western blot, as determined by the relative expression to the housekeeping control vinculin. Bar graph showing the results from two independent experiments; error bars represent SEM.
f) qRT-PCR analysis of *UGP2* in NSCs or KO NSCs rescued with either the long wild type or long mutant UGP2 isoform. Mean fold change compared to wild type is shown for two biological replicates and three technical replicates; error bars represent SEM.

Supplementary Figure 5: RNA-seq, related to Figure 4

a) Scatter plot showing the pair wise correlation between biological replicates.
b) Heat map displaying Pearson correlation between biological replicates.

c) Table summarizing up- (FDR<0.05 and LogFC>1) and down regulated (FDR<0.05 and LogFC<-1) genes in WT, KO and KI ESCs.
d) Table summarizing up- (FDR<0.05 and LogFC>1) and down regulated (FDR<0.05 and LogFC<-1) genes in WT, KO and KI ESC upon differentiation in NSCs.
e) Table summarizing up- (FDR<0.05 and LogFC>1) and down regulated (FDR<0.05 and LogFC<-1) genes in WT, KO and KI NSCs.
f) Heat map visualizing gene expression (in log2(RPKM+1)) and clustering of WT, KO and KI ESCs and NSCs, for a panel of ESC and NSC specific genes (see methods)

Supplementary Figure 6, related to Figure 4: UGP2 mutant induced pluripotent stem cells

a) Immunofluorescence of iPSC clones used in this study derived from Family 1 (three clones per individual) showing iPSC colonies stained for the pluripotency markers TRA1-81 (red) and OCT4 (green) (left panel) or SSEA4 (red) and NANOG (green) (right panel). Nuclei are stained with DAPI (blue).
b) qRT-PCR expression analysis for the indicated pluripotency associated genes in 4 wild type control human embryonic stem cell lines and the iPSCs derived from family 1. Mean fold change compared to human embryonic stem cells of three biological replicates (e.g. individual clones from **a**) and three technical replicates is shown; error bars represent SEM. No statistically significant differences were found, unpaired t-test, two-tailed.
c) Sanger sequencing of representative iPSC clones confirming the presence of the chr2:64083454A>G *UGP2* mutation in a heterozygous state in clones derived from parents and homozygous state in clones derived from the affected child.
d) qRT-PCR PCR expression analysis upon differentiation for pluripotency (*NANOG*, *OCT4* (*POUF51*), *REX1*) and NSC markers (*PAX6*, *GFAP*), for H9 ESC control and heterozygous and homozygous iPSCs derived from family 1. Mean fold change compared to human embryonic stem cells of three biological replicates (e.g. individual clones from **a**) and two technical replicates is shown; normalized to *TBP*; error bars represent SEM.
e) qRT-PCR expression analysis in iPSC-derived NSCs for genes that showed differential expression in RNA-seq experiments, e.g. *NNAT*, *FGFBP3*, *ID4* and *PLAU*. Mean fold change for cells obtained from the affected child compared to cells obtained from its parents (set to 1) of three biological replicates (e.g. individual clones from **a**) and two technical replicates is shown; normalized to *TBP*; error bars represent SEM.

Supplementary Figure 7: related to figure 5

a) UGP2 enzymatic activity in WT, UGP2 KI, KO and KO ESCs rescued with wild type isoform 1 or mutant Met12Val isoform 1 of UGP2. Plotted is the mean from two replicate experiments, error bar is SEM. ***=p<0.001, unpaired t-test, two-tailed.
b) UGP2 enzymatic activity in iPSC derived NSCs from family 1. Plotted is the mean from two replicate experiments, measuring each the results for the three clones for each individual, error bar is SEM. *=p<0.05; unpaired t-test, two-tailed.
c) PAS staining in WT and UGP2 KO ESCs. Nuclei are counterstained with hematoxylin (blue).
d) Quantification of the PAS stained area in WT, KI and KO ESCs. Shown is the average PAS positive area per genotype from two biological replicates, each stained in two experiments; error bars are SD. ***=p<0.001, unpaired t-test, two-tailed.

e) Glycogen granules detected by PAS staining in iPSC-derived NSCs from family 1 after 48 hours culture under low-oxygen conditions. Number of granules for paternal cell line are set at 100%. Average of three biological and two technical replicates per genotype, with each n=80-100 cells counted. Error bars represent SD, ***=p<0.001, unpaired t-test, two-tailed.

f) Western blotting detecting LAMP2 (upper panel) and the house keeping control actin (lower panel) in cellular extracts from ESCs, that are WT, UGP2 KI, or KO. Compare to Figure 5D.

g) qRT-PCR expression analysis for UPR marker genes (spliced *XBP1*, *HSPA5*, *ATF4* and *EDEM*) in WT, UGP2 KI, KO and rescue ESCs. Shown is the mean fold change for the indicated genes compared to wild type, normalized for the housekeeping gene *TBP*. Results of two biological and three technical replicates are plotted from two experiments. Error bars represent SEM; *= p<0.05, unpaired t-test, two-tailed).

h) qRT-PCR expression analysis for UPR marker genes (spliced *XBP1*, *HSPA5*, *ATF4* and *EDEM*) in in primary fibroblasts from family 1. Shown is the mean fold change for the indicated genes compared to wild type, normalized for the housekeeping gene *TBP*. Results of two experiments with each three technical replicates are plotted. Error bars represent SEM; *= p<0.05, unpaired t-test, two-tailed.

Supplementary Figure 1

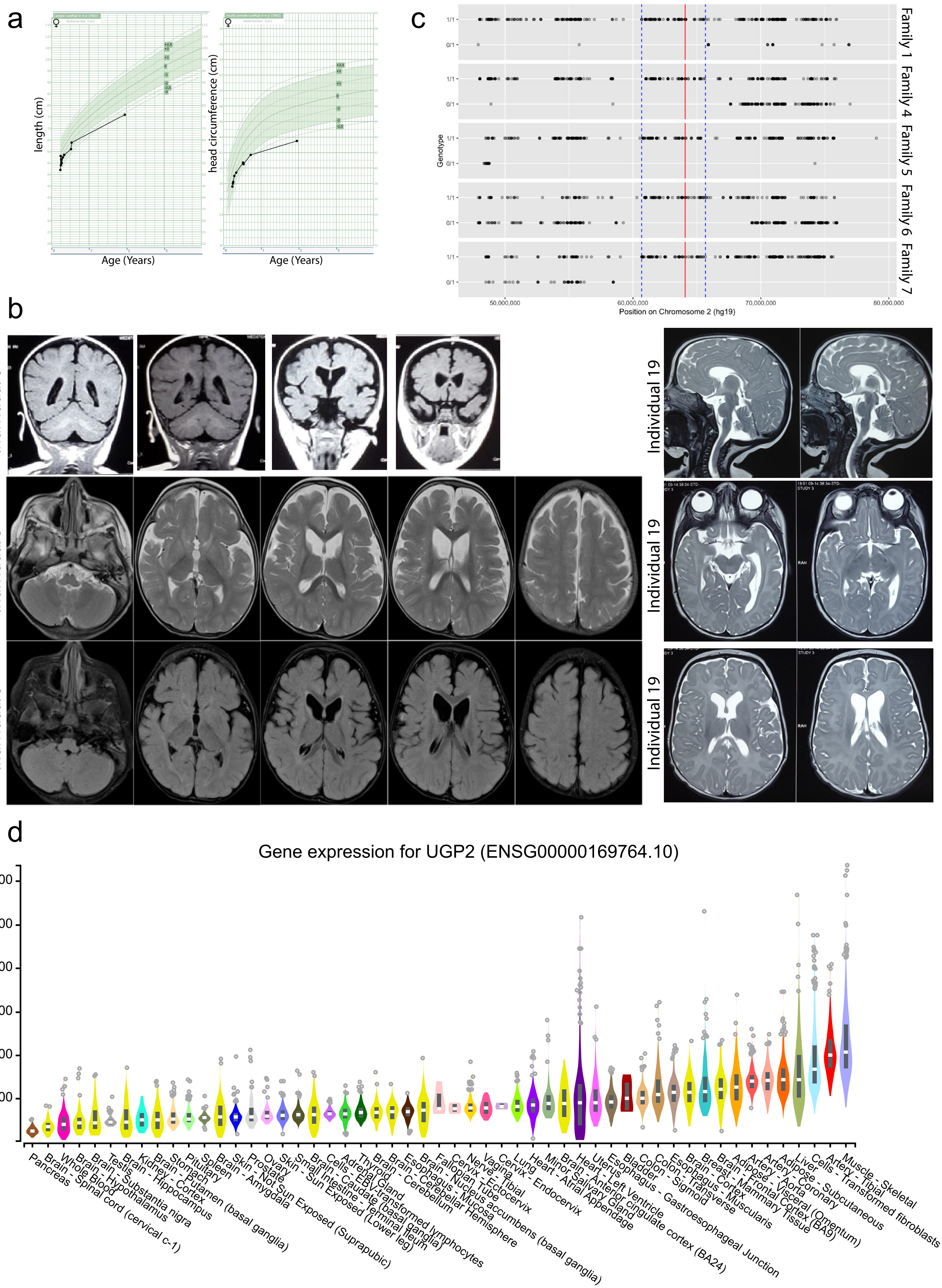

Supplementary Figure 2

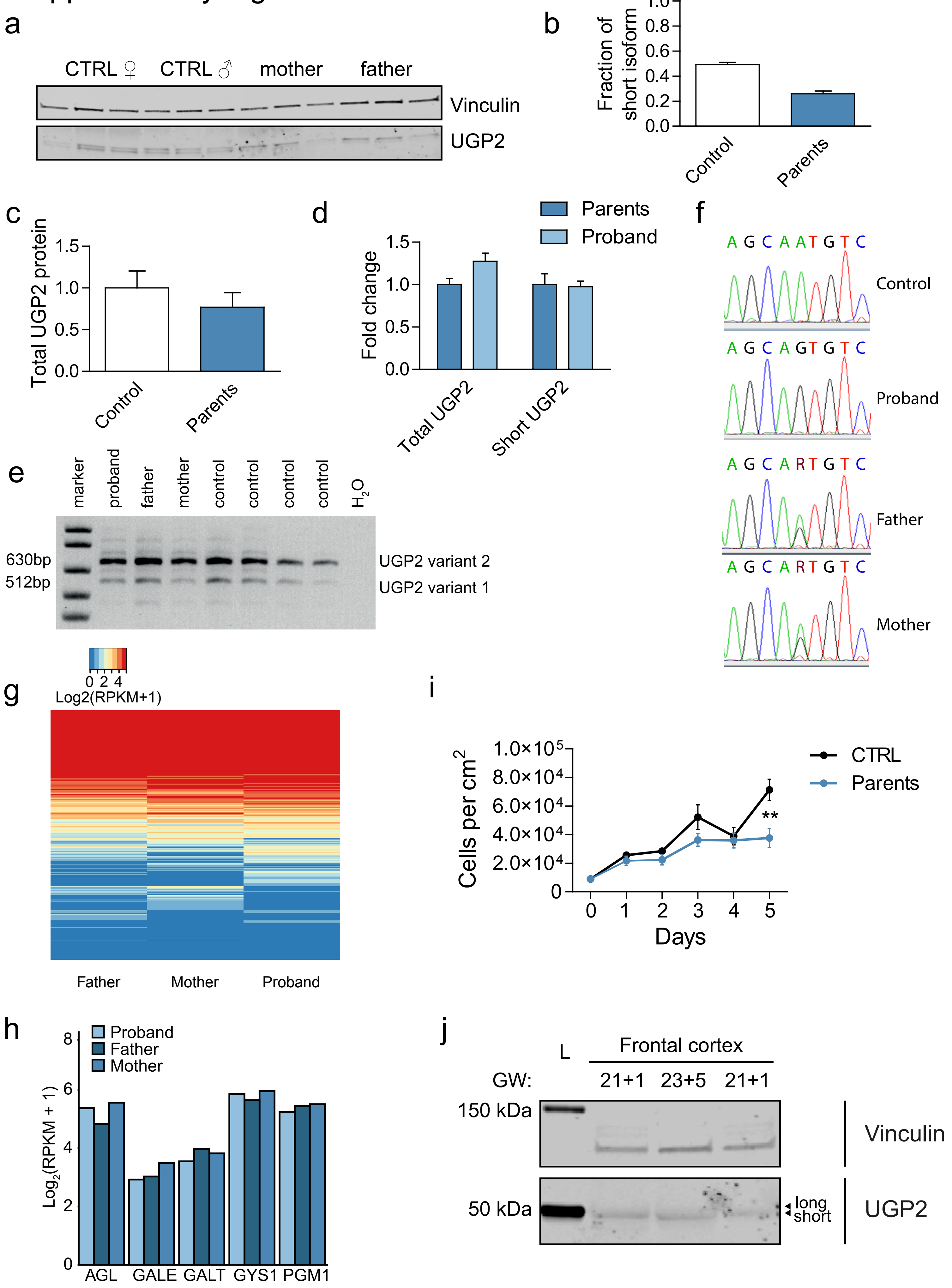

Supplementary Figure 3

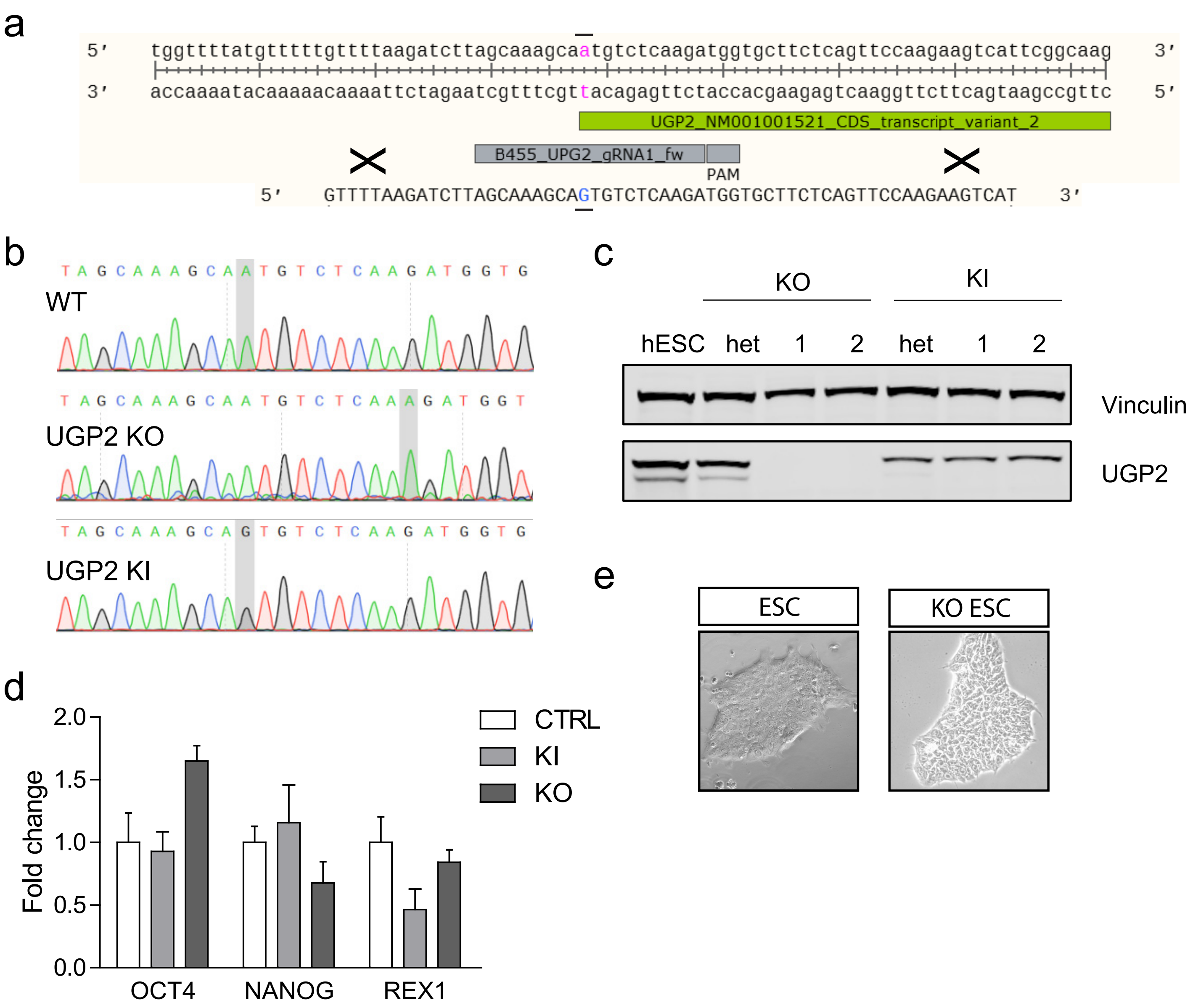

Supplementary Figure 4

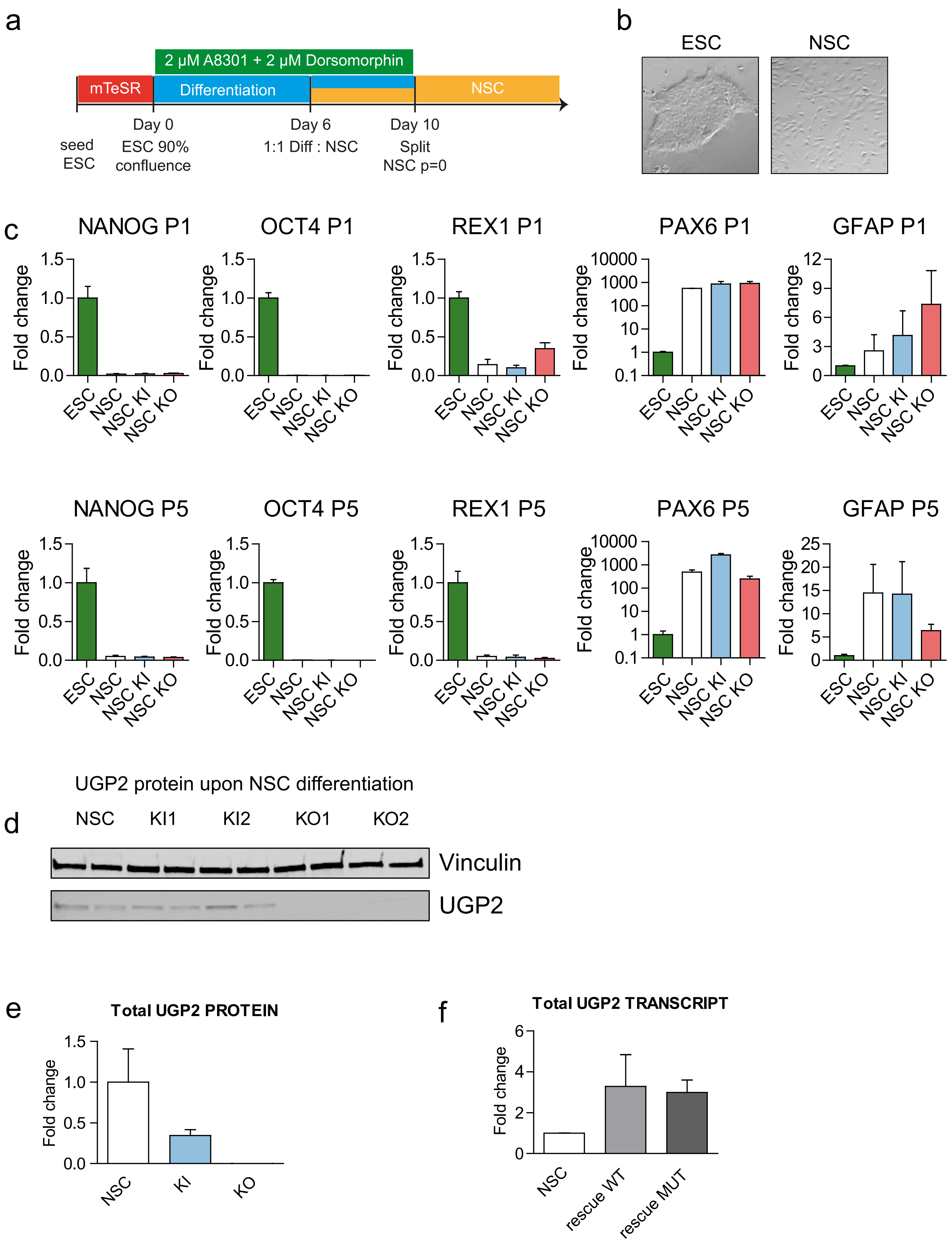

Supplementary Figure 5

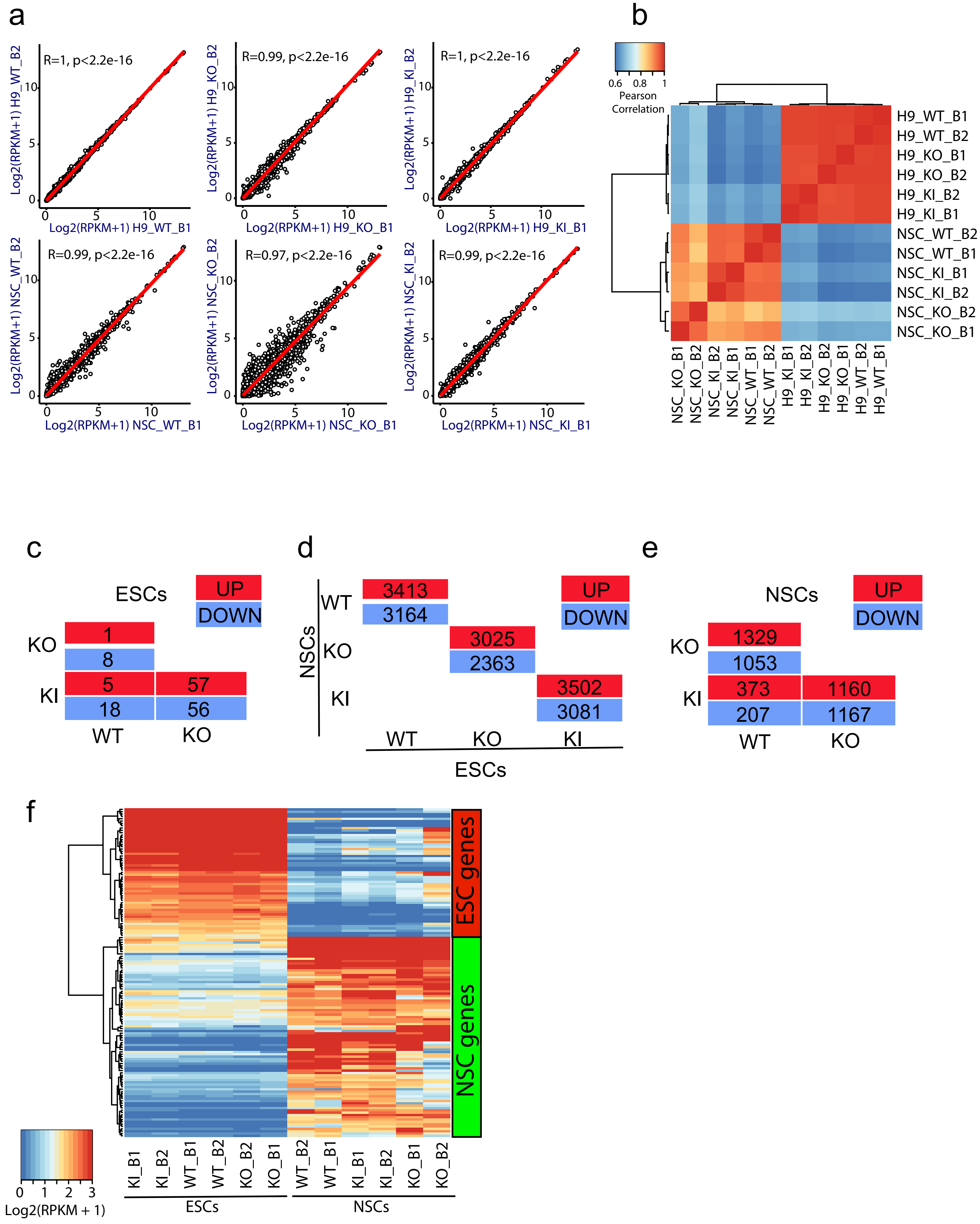

Supplementary Figure 6

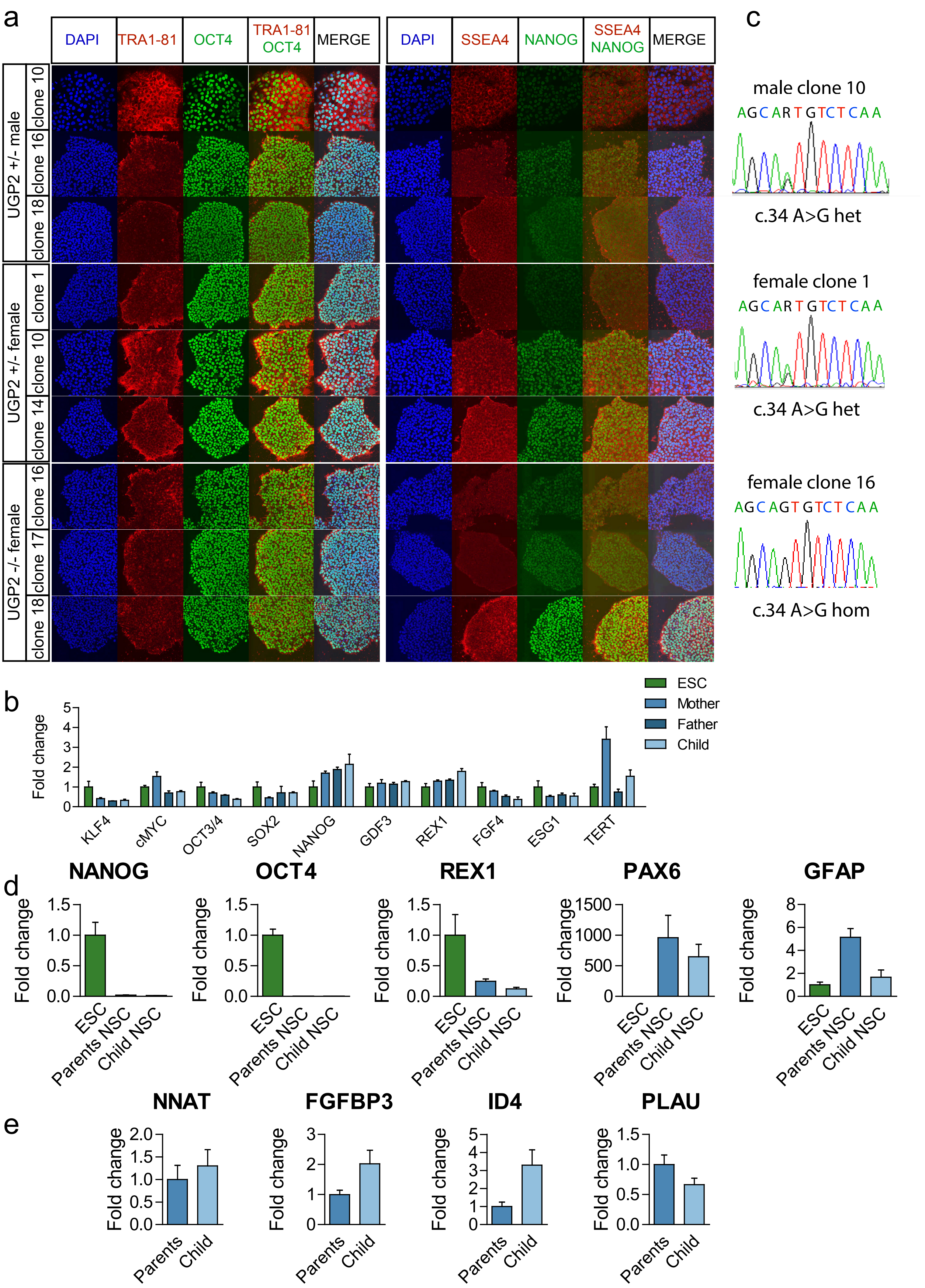

Supplementary Figure 7

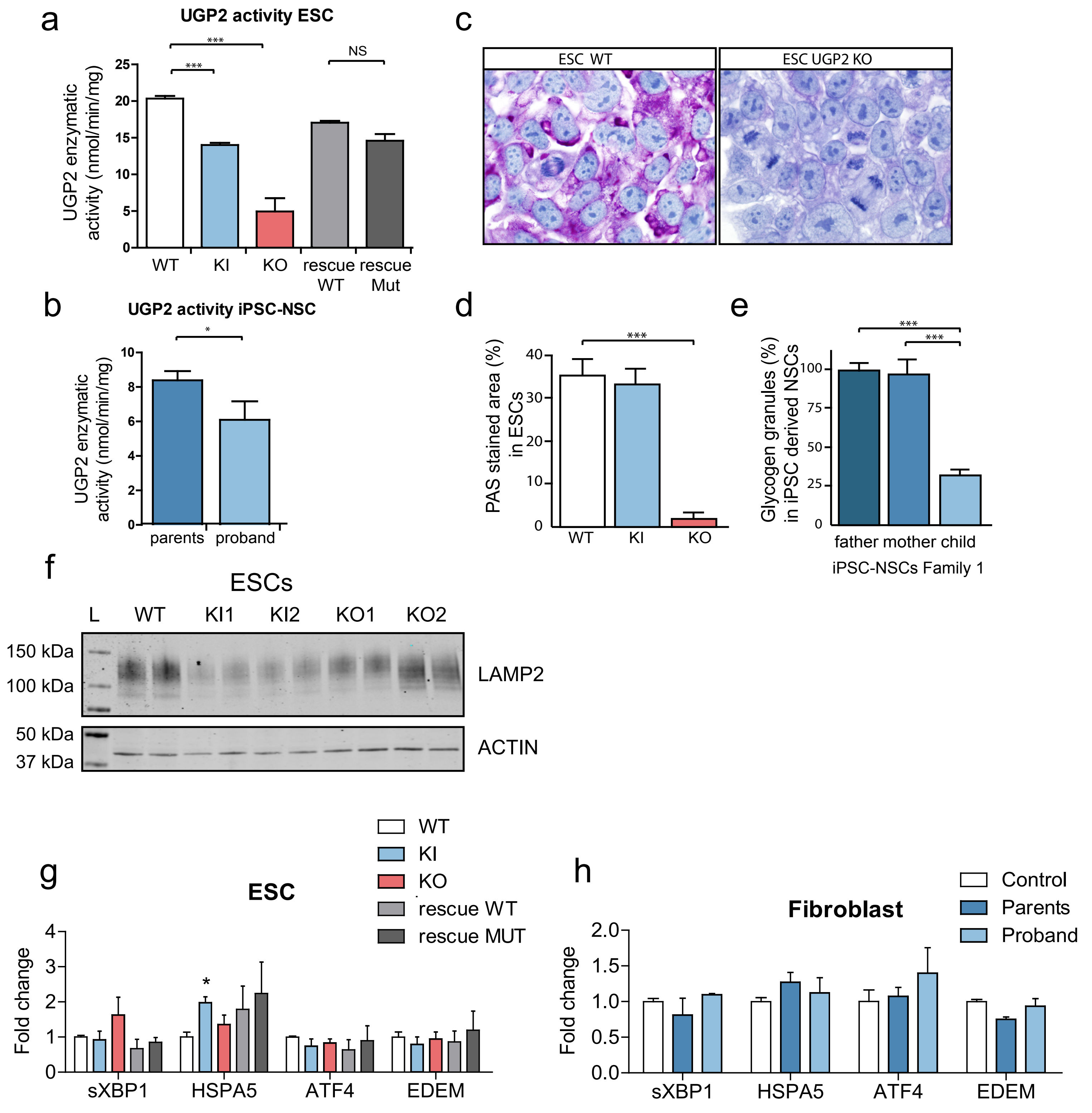

Supplementary Case Reports

Individual 4:

The patient was born at 36+4 weeks after pregnancy complicated by maternal cholestasis. Her parents are of Indian ancestry. There is no recognized consanguinity. The patient was diagnosed with beta thalassemia in the newborn period which required regular transfusions. Feeding difficulties were also noted in the newborn period and persisted. Gastrostomy feeding was initiated at 7 months of age. Seizures were first observed at 3 months of age. The seizures were initially myoclonic and hypsarrhythmia was seen on EEG. The patient’s epilepsy had been intractable and over time she has demonstrated a variety of seizure types including hemiclonic, focal motor, generalized tonic-clonic and tonic. A trial of the ketogenic diet was not effective. Multiple anti-epileptic drugs have been used with limited improvement of seizure frequency. Her primary regimen consisted of phenobarbital and clonazepam. Beginning at age of 10 months the patient began to have severe, dystonic episodes that featured posturing and variation in heart rate. The was also diagnosed with and treated for intussusception at this time. The dystonic episodes improved some with the administration of clonidine and propranolol. Benzodiazepines and opioids were not effective. MRI of the brain was performed at ages 1, 2 and 3 years. A thin corpus callosum was noted and over time there was cortical and striatal volume loss. She has been diagnosed with cortical visual impairment. Eye exam noted lagophthalmos and mild disc pallor. Her linear growth and weight were typical for age. She was able to vocalize but did not achieve other developmental milestones before she passed at age 3.5 years.

Individual 5:

A 9-year-old female child from Oman, who presented at the age of 10 weeks with one day history of recurrent episodes of generalized tonic clonic seizures. She was born to first degree consanguineous parents at full-term, via spontaneous vaginal delivery with a birth weight of 2860 grams and an Apgar of 7 and 10 at 1 and 5 minutes respectively. She is the 5th child for the parents and one of her elder siblings died at 4years of age with some brain malformation (No documents available) and all other siblings are normal except a boy who reportedly has intellectual disability. Her clinical examination, on initial admission showed a head circumference of 37 cm (between 50th and 75th centiles) with weak Moro and sucking reflex, power of 4/5 on the limbs and exaggerated deep tendon reflexes. All the baseline investigations were within normal limits and she was loaded with phenobarbitone and continued with a maintenance dose. As the seizures were not well controlled, she required phenytoin, levetiracetam, topiramate and midazolam infusion during the first admission.

Her seizures got controlled after she was started on midazolam infusion. Her EEG at that time showed multifocal seizures with burst suppression and MRI brain showed cerebral atrophy with a thin corpus callosum and delayed myelination. An oral pyridoxine trial was started and she was referred to for further metabolic work-up. She was seen by a metabolic consultant, but her parents refused further investigations at that time and went against medical advice. During a second opinion in Pakistan she was started on ACTH for 6 weeks but this did not result in improvements. Parents stopped phenytoin treatment after hospital discharge and the child continued to get daily recurrent episodes of multiple types of seizures (generalized tonic clonic, tonic seizures, flexor spasms).

After 4 months parents visited again our outpatient clinic and at that time the girl had not attained any head control, did not visually track and had bilateral pyramidal signs. She was on phenobarbitone, topiramate and levetiracetam at that time. After adjustments of medication doses, clonazepam was added, which resulted in a slightly reduced seizure frequency. Her ophthalmic assessment showed generalized disc pallor with severe visual impairment. During her follow up as the seizures were not well controlled, she was started on trial of folinic acid and parents felt that the seizures improved after starting folinic acid. Parents noticed that seizure frequency had increased while they ran out of folinic acid for a week. During the follow-up, she was admitted twice to complete the detailed metabolic work ups.

Relevant investigations:

FBC - Normal
Bone profile, Electrolytes, LFT, Magnesium: Normal
Ammonia: 50 umol/L
Lactate: 1mmol/L
Blood gas: Normal
Tandem Mass Spectrometry: Unremarkable
Uric acid: 0.20 mmol/L (0.15 -0.35)
Urine organic acids: unremarkable
Lysosomal enzymes: unremarkable
Serum pyridoxal phosphate: 206 nmol/L (35 -110)
Plasma homocysteine: 7 umol/L (< 10)
Urine sulfocystiene: Not detected
Plasma amino acids: Unremarkable
CSF Lactate: 1.6 mmol/L
CSF Glucose: 3mmol/L (Blood glucose -5 mmol/L)
CSF Amino acids -Slight decrease in Glycine (4,0 µmol/l Reference values 6.0-11.0) moderate increase in glutamine (606,0 µmol/l Reference values 333.9-575.5)
CSF biogenic amines: Normal
Serum Pipecolic acid: Normal
CDG (Congenital disorder of glycosylation): Normal
EEG: Abnormal for frequent generalized spike and wave discharges followed by brief period of suppression of background. Also independent epileptiform discharges arising from both temporal regions which become almost continuous at times. Also noticed to have asynchrony. The EEG is suggestive of early epileptic encephalopathy.
MRI Brain: Cerebral atrophy with thin corpus callosum and delayed myelination
MRI Brain: Generalized brain atrophy more marked in the supratentorial compartment with scanty white matter
USG Abdomen: Normal

Last clinical review: She was still having daily brief seizures on multiple occasions. She had not attained any developmental milestones She is on nasogastric feeding with formula milk only. Examination showed a bedridden child with microcephaly, no vision and hearing, no facial asymmetry, generalized hypotonia with grade 3/5 power in both upper and lower limbs, DTR are just elicitable, and planters are -flexor bilaterally. Current medications: Calcium Folinate 5mg BID, Phenobarbitone 30 mg BID, which is 4.3 mg/kg/day Topiramate 25mg am and 50mg pm which is 5.4 mg/kg/day, Levetiracetam 250 mg BID, which is 36 mg/kg/day, Clonazepam 300mcg BID.

Individual 12:

Individual 12 was born at term with unremarkable perinatal history. Growth parameters were normal. The parents were first-degree cousins. Two maternal uncles had global delay with intractable epilepsy and died at age of 1 and 4 years, respectively. At three months, the baby was noted to have episodic leg jerking which was confirmed to be epileptic seizures. With time, seizures became more frequent and daily, consisting of brief tonic seizures with uprolling of eyes. Several combinations of antiepileptic drugs were tried, but seizures remained intractable. The latest of which included phenobarbital, topiramate, and levetiracetam. Trial of pyridoxine was not helpful.

Comprehensive metabolic investigations were unrevealing. These included serum lactate, amino acids, renal and hepatic profiles, ammonia, transferrin isoelectric focusing, acyl carnitine profile and urine organic acids. EEG showed frequent generalized spikes during sleep associated with frequent independent sharp waves over frontal and central areas bilaterally. Trial of steroids – suspecting variant Landau Kluffner syndrome-was not helpful either. Brain MRI showed brain atrophy and developmental changes in the mesial temporal lobes. Long bone and chest X-rays showed osteopenia, leading to one event of femoral fracture. No abnormal storage was noted in skeletal bones or on femur MRI. Abdominal ultrasound showed borderline liver size but normal echogenicity. Thigh Muscle MRI showed possible moderate diffuse fatty changes involving both gluteal muscle groups and posterior thigh muscle compartment in both sides, with milder fatty changes in the anterior thigh compartment. Currently, at age 10, he is stroller bound, profoundly globally delayed in development. He is fed through nasogastric tube due to severe dysphagia. No organomegaly or major dysmorphic features are noted. His seizures are tonic, brief lasting seconds with up-rolling of eyes that happen daily, sometimes triggered by sound. They are more frequent upon awaking. He is not attentive to parents, both with sound or visual stimulation. Flash VEP showed delayed p100 wave and an abnormal electroretinogram. He is on multiple antiepileptic drugs including, toperamate, levetiracetam and phenobarbital as well as pyridoxine.

Individual 13:

Individual 13 is the affected sister of individual 12. She was born at term with unremarkable perinatal course and normal birth growth parameters. The mother noticed seizures at the age of 5 months which were having semiology of infantile spasm, with flexion of the trunk and the upper limbs. Attacks were occurring in clusters. She was noted to be developmentally delayed as she was unable to support her neck when she was first evaluated at the age of 7 months. When examined, height, weight and head circumferences were between 10th and 50th percentiles. She was spastic with brisk reflexes. The rest of systemic examination was normal. MRI showed prominence of bilateral frontal horns with brain atrophy. EEG was abnormal showing paroxysmal epileptiform discharges but no classical hypsarrythmia. Brain auditory evoked potentials, electroretinography and visual evoked potentials of the left eye were normal while visual evoked potentials of the right eye showed reduced amplitude of p100. Comprehensive metabolic testing with serum, urine and CSF analysis were unrevealing. CSF/serum glucose ratio was normal excluding possibility of Glut-1 deficiency. WBC Electron microscopy for neuronal ceroid lipofuscinosis was negative. The patient was severely handicapped and seizures were difficult to control. She was treated with pyridoxine, levetiracetam and vigabatrin. At the age of 15 months, she died when she had a febrile illness with increased seizures. The cause of death was presumed aspiration with respiratory arrest at home.

After finding UGP2 as the main candidate gene for both affected siblings, the parents of family 10 elected to pursue preimplantation genetic diagnosis and in-vitro-fertilization upon genetic counseling. Following controlled ovarian stimulation, fourteen oocytes were retrieved and nine were found to be suitable for biopsy on day 3. Karyomapping, haplotype chart and detailed haplotype analysis were reviewed and risk of contamination was excluded using AmpFlSTR® Identifiler® PCR Amplification Kit (following the manufacturer's instructions). Two embryos were selected for transfer, embryo #2 and #5. Genetic analysis indicated that embryo 2 is a carrier with the inheritance of the normal maternal allele whereas embryo 5 showed completely normal pattern. Both embryos were chromosomally normal (euploid) and resulted in the delivery of normal born twin (carrier male and normal female). Currently at 25 month both children are free from any disease symptoms.

Individual 20:

2 year old male. Regression from 3 month of age with neurodevelopmental delay. Focal onset-seizures, generalized seizures, epileptic encephalopathy. Abnormal EEG Hypsarrhythmia. Brain MRI showing brain atrophy. Consanguineous parents. 2 affected sisters deceased at 8 days and 1 year.

WES identified 2 homozygous pathogenic variants in TTLL5 and ARMC4, consistent with a genetic diagnosis of autosomal recessive cone-rod dystrophy type 19 and autosomal recessive primary ciliary dyskinesia type 23. None of these however explain the neurological phenotype. Upon re-analysis, the recurrent homozygous variant in UGP2 (UGP2 NM_001001521.1:c.1A>G NM_001001521.1:p.Met1?) was identified. Both parents were heterozygous carriers.

Individual 22:

Female. Epileptic encephalopathy, regression, NDD.

Supplementary Note

The disease we here describe is caused by the loss of an isoform of an essential gene, due to an alteration affecting an isoform specific start codon. To investigate whether this same mechanism could apply to other essential genes that were previously not implicated in human genetic disease, we investigated the occurrence of homozygous or hemizygous ATG altering mutations using data mining of whole exome sequencing data from undiagnosed patients from our own data base, the Queen Square Genomic Center database and those from *Centogene* and *GeneDx,* focusing on the list of genes presented in **Figure 7***.* This identified a number of currently genetically unexplained individuals with homozygous and hemizygous start codon altering variants, that we will report elsewhere in more detail.

We here briefly describe as an additional example of the mutational mechanism the occurrence of a hemizygous start codon altering variant in the peptidylproly cis/trans isomerase, NIMA-interacting-4 gene *PIN4* (NM_006223.3:c.2T>A, p.Met1?). In the *CentoMD* data base, we identified 5 hemizygous patients, presenting with a shared phenotype of neurodevelopmental delay, microcephaly, seizures, inguinal hernia and a few other shared features, that we will describe elsewhere in full detail. Using routine clinical diagnostics, including whole exome and whole genome sequencing, no alternative disease explaining variant has been identified in these individuals.

The variant is absent in *gnomAD*, and not found in our in house data bases. We did not identify any other LoF variant in this gene in our cohorts.

PIN4 encodes a member of the parvulin subfamily of the peptidyl-prolyl cis/trans isomerase family. It catalyzes the isomerization of peptidylprolyl bonds, and is proposed to play a role in cell cycle, chromatin remodeling, ribosome biogenesis and mitochondria function. Importantly, it has been shown to influence the formation of microtubules [2]. PIN4 is widely expressed amongst tissues, including different brain regions, according to data from the GTEX portal (**Figure**)[1]. Together, this makes *PIN4* a strong candidate gene for a novel neurodevelopmental disorder.

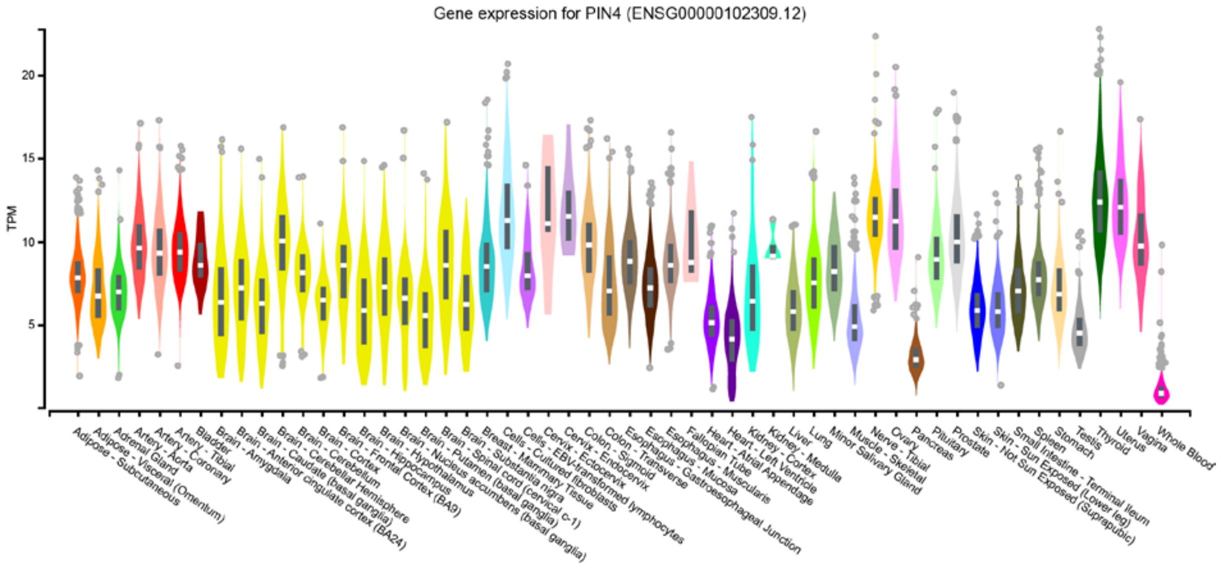

Supplementary References

1 Consortium GT (2013) The Genotype-Tissue Expression (GTEx) project. Nat Genet 45: 580-585

2 Thiele A, Krentzlin K, Erdmann F, Rauh D, Hause G, Zerweck J, Kilka S, Posel S, Fischer G, Schutkowski Met al (2011) Parvulin 17 promotes microtubule assembly by its peptidyl-prolyl cis/trans isomerase activity. J Mol Biol 411: 896-909
